# Functional plasticity in oyster gut microbiomes along a eutrophication gradient in an urbanized estuary

**DOI:** 10.1186/s42523-020-00066-0

**Published:** 2021-01-06

**Authors:** Rebecca J. Stevick, Anton F. Post, Marta Gómez-Chiarri

**Affiliations:** 1 Graduate School of Oceanography, University of Rhode Island, Narragansett, RI USA; 2 Division of Research, Florida Atlantic University, Boca Raton, FL USA; 3 Department of Fisheries, Animal and Veterinary Sciences, University of Rhode Island, Kingston, RI USA

**Keywords:** Microbiome, 16S rRNA gene sequencing, Metatranscriptomics, Coastal eutrophication, Oysters, *Crassostrea virginica*

## Abstract

**Background:**

Oysters in coastal environments are subject to fluctuating environmental conditions that may impact the ecosystem services they provide. Oyster-associated microbiomes are responsible for some of these services, particularly nutrient cycling in benthic habitats. The effects of climate change on host-associated microbiome composition are well-known, but functional changes and how they may impact host physiology and ecosystem functioning are poorly characterized. We investigated how environmental parameters affect oyster-associated microbial community structure and function along a trophic gradient in Narragansett Bay, Rhode Island, USA. Adult eastern oyster, *Crassostrea virginica,* gut and seawater samples were collected at 5 sites along this estuarine nutrient gradient in August 2017. Samples were analyzed by 16S rRNA gene sequencing to characterize bacterial community structures and metatranscriptomes were sequenced to determine oyster gut microbiome responses to local environments.

**Results:**

There were significant differences in bacterial community structure between the eastern oyster gut and water samples, suggesting selection of certain taxa by the oyster host. Increasing salinity, pH, and dissolved oxygen, and decreasing nitrate, nitrite and phosphate concentrations were observed along the North to South gradient. Transcriptionally active bacterial taxa were similar for the different sites, but expression of oyster-associated microbial genes involved in nutrient (nitrogen and phosphorus) cycling varied throughout the Bay, reflecting the local nutrient regimes and prevailing environmental conditions.

**Conclusions:**

The observed shifts in microbial community composition and function inform how estuarine conditions affect host-associated microbiomes and their ecosystem services. As the effects of estuarine acidification are expected to increase due to the combined effects of eutrophication, coastal pollution, and climate change, it is important to determine relationships between host health, microbial community structure, and environmental conditions in benthic communities.

**Supplementary Information:**

The online version contains supplementary material available at 10.1186/s42523-020-00066-0.

## Background

Coastal ecosystems serve as habitat for highly diverse communities that contribute up to 77% of worldwide ecosystem services [1, 2]. Humans directly rely on these environments for activities like tourism and fisheries, and for their ecosystem services [3–5]. Environmental conditions in coastal estuarine ecosystems fluctuate rapidly due to changes in nutrient loading, river runoff, and other physical, chemical, and biological factors [6–8]. For example, average pH values in coastal waters can vary by as much as one pH unit over both daily and seasonal cycles, reflecting changes in biological outputs like microbial activity, ambient dissolved oxygen (DO), and pCO_2_ [9, 10]. These frequent changes in estuarine water chemistry are also affected by human activity and coastal geomorphology, and these influences will likely increase over the coming decades [11].

Estuaries such as Narragansett Bay, Rhode Island, USA, provide a natural gradient to study the impacts of eutrophication. The head of the Bay, located in a highly urbanized area, is highly eutrophic while trophic levels at the mouth are more similar to those found over the continental shelf [12, 13]. Previous studies have shown that eutrophication in the headwaters of Narragansett Bay affects many local communities and ecosystem processes at locations downstream, including nitrification rates [14], primary productivity [15], animal physiology, and benthic biodiversity [16, 17]. Over the last 20 years, Narragansett Bay has undergone dramatic changes as a result of targeted efforts to reduce nutrient loading, providing a dynamic model for the study of gradients in estuarine eutrophication [18].

Marine microbial communities play a central role in ecosystem function as the engine for carbon and nutrient cycling. Microbial communities in coastal seawater and sediment exhibit plastic responses to environmental changes or gradients [19–22]. This may lead to changes in primary productivity, and therefore coastal ecosystem functioning [23]. Studies of bacterial community structures and nitrogen cycling in several coastal lagoons found that physical gradients and nutrients affect sediment microbial interactions and function [19, 24]. In marine sediments exposed to high nutrients, studies reported dramatic changes in ecological function, but no significant differences in microbial community structure [25–27].

Host-associated microbiomes are gaining importance as major contributors to ecosystem services and host functioning [28, 29]. Various studies have found that environmental conditions affect microbial community structures in marine hosts, including corals [30], sponges [31], eelgrass [32], seagrass [33], oysters [34] and mussels [35]. Varying pollution levels alter Manila clam, *Ruditapes philippinarum,* microbiome composition and host susceptibility to chemicals, as well as kelp bacterial community composition [36, 37]. Studies that examine host-associated microbial functional responses to environmental change, however, are limited to survey studies and focus on model organisms (i.e. corals or zebrafish) in lab-based studies [38]. For example, a study of foundational corals found that nutrients did not affect the host fitness or health, but caused shifts in certain microbial taxa that may influence microbial function [30].

In Narragansett Bay, as in other temperate coastal estuaries, the eastern oyster, *Crassostrea virginica*, is an integral part of the local history, culture, and seafood industries. Moreover, oysters provide many ecosystem functions, including clearing of overlying waters, coastal erosion prevention, and nutrient cycling [39]. Oyster-associated microbiomes significantly contribute to these ecosystem services, as the oyster host retains and provides a habitat for specific bacteria that perform denitrification and assimilate excess phosphorus [40, 41]. Microbes may also aid in maintaining oyster health and homeostasis by controlling infection, performing nutrient removal, or providing metabolites [42–44].

Previous studies of microbial ecology in oysters and other hosts have been limited to surveys of microbial community structures in different compartments of the host. There are, however, very few studies investigating the impact of environmental change on the function of host-associated microbiomes, and how those functional changes may affect coastal ecosystem function. The microbiomes of adult oysters, as determined by 16S rRNA gene amplicon sequencing or other genetic markers, vary with location [45, 46], season [34], tissue type [47], disease status [43, 48, 49], and environmental conditions [50]. Some studies have attempted to infer oyster-associated microbial function from 16S rRNA gene amplicon sequencing, but this method relies on phylogenetically conserved function and is largely speculative [51, 52]. In this study, we evaluated the structure and function of eastern oyster, *C. virginica,* gut microbiomes at five sites along the eutrophication gradient in Narragansett Bay using 16S rRNA gene amplicon sequencing and metatranscriptomics. This survey provides a snapshot of the oyster microbiomes in a relatively small geographic area in a temperate coastal estuary affected by eutrophication, and how these host-associated microbiomes are affected by their local environment.

## Results

### Sampled sites showed variability in environmental conditions

**Fig. 1:**
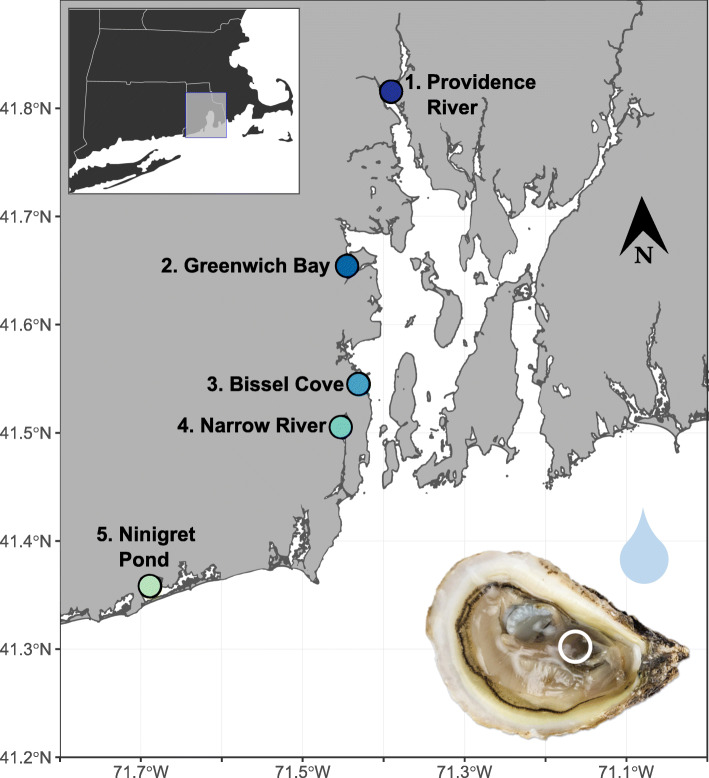
Map of study area with 5 sampling locations. A schematic of the samples collected from each site is shown in the bottom right

**Fig. 2:**
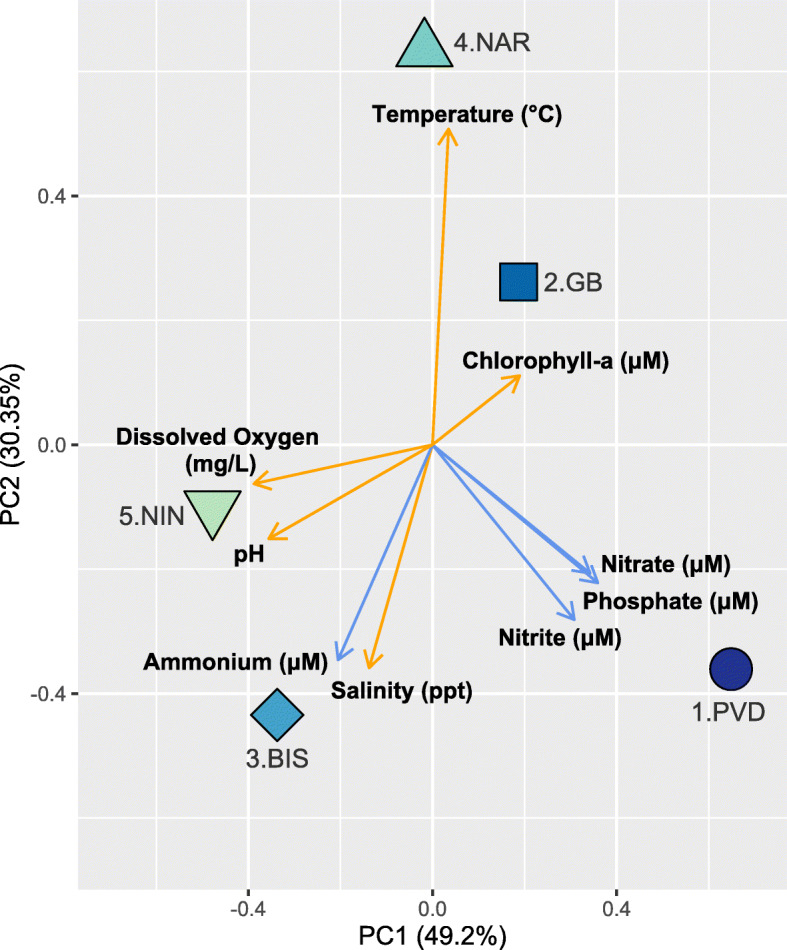
Environmental conditions characterizing each site. Principal component analysis of each site is represented by a colored symbol and each environmental factor is represented with an arrow. Orange arrows indicate average environmental values measured in situ during the sampling week (*n* = 2); light blue arrows represent nutrient concentrations measured from water samples (*n* = 3)

Five sites were selected along Narragansett Bay: 1.PVD (Providence River: Bold Point Park), 2.GB (Greenwich Bay: Goddard Memorial State Park), 3.BIS (Bissel Cove: Rome Point), 4.NAR (Narrow River), and 5.NIN (Ninigret Pond) (Fig. 1). These sites are representative of a diversity of environmental conditions (i.e. nutrients, dissolved oxygen, pH, salinity) within a coastal estuary and varying levels of anthropogenic inputs (Table 1). A North-South estuarine gradient was detected, especially in nutrient concentrations. Salinity, pH, and DO increased down the Bay from Providence (1.PVD; North) to Ninigret Pond (5.NIN; South), as coastal eutrophication and the influence of river inputs decreased (Table 1, Spearman’s Correlation Coefficients, SCC = -0.8, − 0.8, − 0.9). Nutrient concentrations decreased along the North-South gradient (Table 1, SCC = 0.7, 0.6, 0.9), with 1.PVD showing significantly higher concentrations of nitrite, nitrate, and phosphate than all other sites (*p* < 0.01). A Principal Component Analysis (PCA) of these measured environmental conditions (each averaged per site), with eigenvalues in components one and two representing 80% of the environmental variation between sites is shown in Fig. 2. Each site was characterized by a subset of environmental factors over the sampling period. The 1.PVD site was characterized by the highest nutrient levels (nitrite, nitrate, phosphate, *p* < 0.01, compared to all other sites), 2.GB by the highest chlorophyll-a, 3.BIS by the highest ammonium concentrations (*p* < 0.001), 4.NAR by a higher temperature (NS) and significantly lower salinity than all other sites (*p* = 0.045), and 5.NIN by significantly higher pH than all other sites (*p =* 0.023). The average mass, length, and width of oysters at each site decreased down the Bay, with the exception of oysters from 3.BIS, which were significantly heavier and larger than oysters collected at other sites (Table 1, SCC = 0.7,0.7,0.8; *p* < 0.001).

**Table 1:**
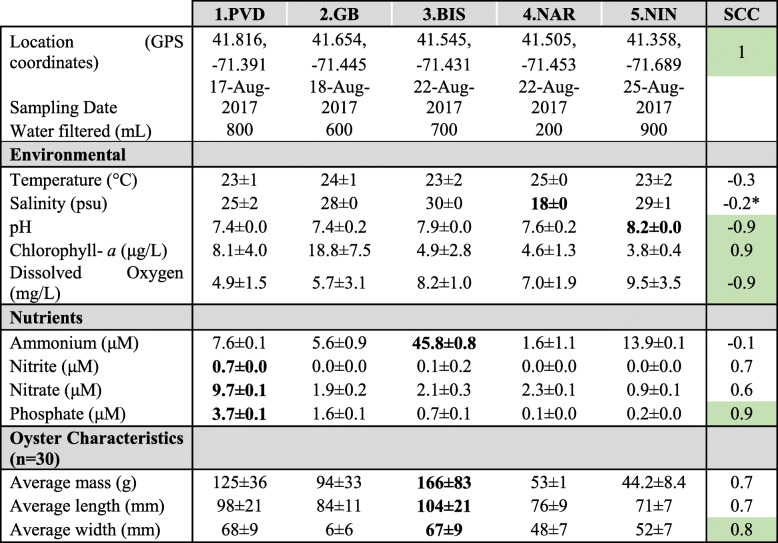
Summary of all measurements collected per site. Environmental values are daily averages ± standard deviation measured at each site 1 day during week of collection. Nutrient values are averages of three-point water samples collected from each site at time of oyster collection. Spearman’s correlation coefficient (SCC, − 1 to 1) was calculated for the association between each parameter and Latitude. The most significant SCC values (| ≥ 0.8|) are shaded green. A value closer to 1 indicates that the parameter decreases from North-South (1.PVD to 5.NIN) and a value closer to − 1 indicates that the parameter increases from North-South. A correlation coefficient of 0 means there is no linear association and that the value does not consistently change along the estuarine gradient. Significant values as compared to the other sites are indicated in bold. *Spearman’s correlation coefficient for Salinity without 4.NAR: -0.8

### Differences in microbial community structures were observed between sites and sample types

**Fig. 3:**
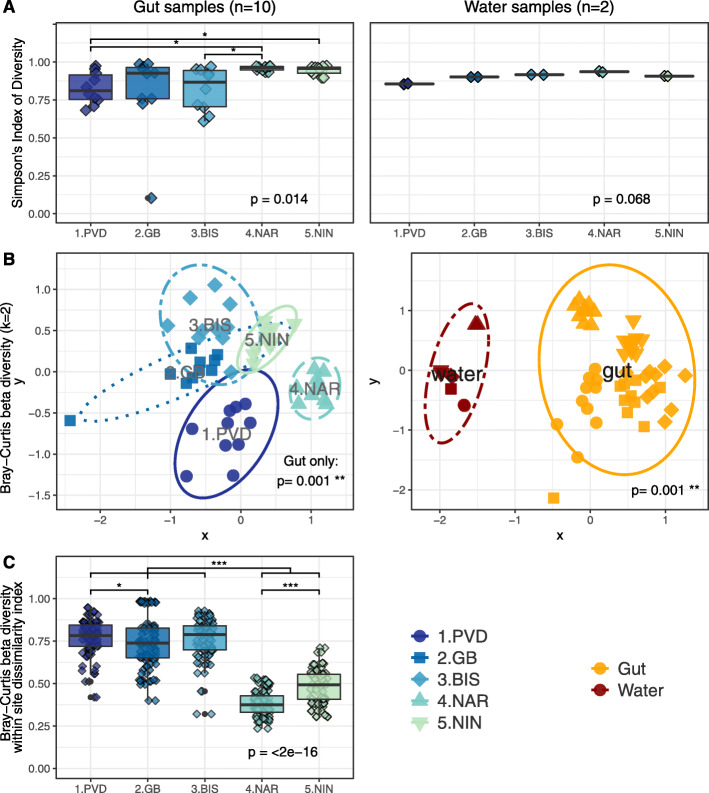
Effect of site and sample type on diversity indices in present bacterial community structures. **a** Simpson’s Index of Diversity calculated using ASV-level 16S rRNA gene amplicons for gut samples (left, *n* = 10) and water samples (right, *n* = 2). Global *p*-values were calculated using the Kruskal-Wallis rank-sum test, and pairwise *p*-values were calculated with the Wilcox rank-sum test. **b** NMDS plot visualization of Bray-Curtis beta-diversity (k = 2) at the ASV level for gut samples by Site (left) and all samples by Type (right). The ellipse lines show the 95% confidence interval (standard deviation). *p*-values indicate significance of grouping with adonis2 Permutational Multivariate Analysis of Variance Using Distance Matrices test. **c** Within site Bray-Curtis dissimilarity index values for gut samples (*n* = 90; 9 comparisons for each of 10 samples per site). Global *p*-value was calculated using the Kruskal-Wallis rank-sum test, and pairwise *p*-values were calculated with the Wilcox rank-sum test

**Fig. 4:**
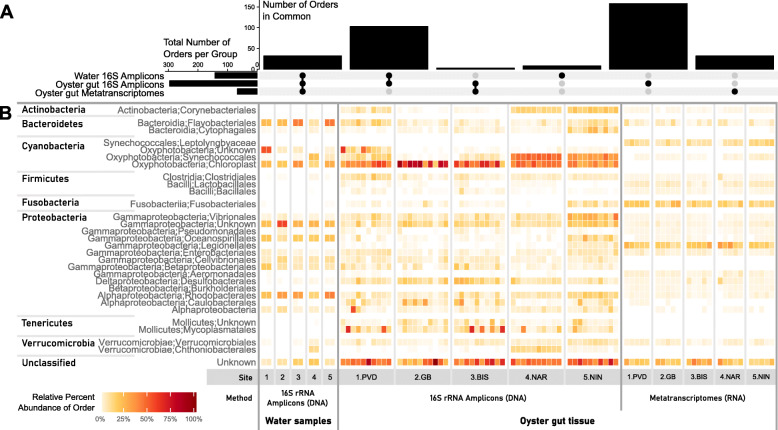
Effect of site and sample type on present and active bacterial community structures. **a** Number of bacterial Orders shared between the water 16S rRNA gene amplicons, oyster gut 16S rRNA gene amplicons, and oyster gut metatranscriptomes (vertical bars). The total number of Orders found in each group is shown in the horizonal bar graph on the left. **b** Relative percent abundances (square root rescaled) of top 30 bacterial Orders associated with seawater samples (*n* = 2) or oyster gut tissue (*n* = 10 or 5), per site. The most abundant bacteria (16S rRNA gene amplicons, middle, *n* = 10) and the most transcriptionally active bacteria (metatranscriptomes, right, *n* = 5) in the oyster gut are shown

A total of 2,217,804 quality-controlled, bacterial 16S rRNA gene sequences were analyzed from 50 gut samples and 10 water samples from 5 sites (Table S1). The sequenced mock community and blank control were analyzed to confirm absence of contamination and adequate sequencing proportions (Fig. S1B). Sequence variant analysis and taxonomic classification resulted in the detection of 304 bacterial Orders across 45 Phyla across all samples, which sufficiently covered the estimated diversity in the samples (Fig. S2). The most dominant phyla in the eastern oyster gut samples, averaged for all oysters at all sites, were *Cyanobacteria* (38 ± 18%) *Proteobacteria* (21 ± 13%), *Tenericutes* (6 ± 12%) and *Actinobacteria* (3 ± 2%) (Fig. S1A). The most dominant phyla in the water column, averaged from all sites, were *Proteobacteria* (62 ± 10%), *Cyanobacteria* (15 ± 12%), *Bacteroidetes* (15 ± 7%), and *Actinobacteria* (3 ± 2%) (Fig. S1A). Differences in bacterial community structures were observed between the oyster gut and water samples, in addition to between sites for both sample types (gut and water) (Figs. 3 and 4, and S3).

### Effect of sample type on microbial community structures

The structure of the gut microbiome was distinct from the water microbial community, regardless of the sampling site (Fig. 3b, stress = 0.19, *df* = 4*,* PERMANOVA R^2^ = 0.46, *p* = 0.001). Of the 304 Orders detected in the 16S amplicon data, the water and gut samples had 135 (45%) Orders in common, while 8 (2%) were exclusively found in the water and 161 (53%) were found only in the oyster gut, suggesting selection by the host (Fig. 4a). LEfSe analysis revealed 22 Orders significantly more abundant in the water than the gut samples, including *Flavobacteriales*, *Rhodobacterales*, *Rhodospirillales*, and *Oceanospirillales* (Figs. 4b and S4A, LDA > 2, *p* < 0.05). Conversely, *Corynebacteriales*, *Propionibacteriales, Desulfobacterales*, and *Mycoplasmatales* were some of the 14 Orders more abundant in gut samples (Figs. 4b and S4A, LDA > 2, *p* < 0.05). Significantly more unknown bacterial Orders were detected in the oyster gut samples, compared to the water (Fig. 4b, t-test *p* < 0.001).

### Effect of site on microbial community structures

The oyster gut bacterial communities from each site were significantly different at the ASV level (Fig. 3b, Table S2, *df* = 4, stress = 0.19, PERMANOVA R^2^ = 0.15, *p* = 0.001). Samples from 1.PVD and 3.BIS showed significantly lower alpha-diversity (Fig. 3a, Simpson’s Index; *p <* 0.01) than samples at other sites. This correlated with specific microbial signatures found at each site. For example, *Corynebacteriales* and *Synechococcales* were significantly more abundant in the gut samples from 4.NAR and 5.NIN than at other sites, and *Verrumicrobia* were significantly more abundant at 4.NAR than in others sites (Figs. 4b and S4B, LDA > 2, *p* < 0.05). The within-site variability of oyster gut microbiomes was significantly higher at the Northern sites (1.PVD, 2.GB, 3.BIS) than at the Southern sites (4.NAR, 5.NIN) (Fig. 3c, Bray-Curtis; *p* = 0.001).

Oyster gut samples at 2.GB showed significantly higher percentages of chloroplast-associated 16S rRNA gene amplicons (50 ± 27%), which is consistent with high chlorophyll-a levels measured at this site (Figs. 2 and S4B, LDA > 2, *p* < 0.05). These sequences were removed prior to calculating dissimilarity metrics. Oyster gut samples from all sites shared 105 Orders (34% of 304 total), while 9–31 (3–10%) Orders were distinct to gut samples at certain sites (Fig. S3).

### Transcriptionally active microbial community structures differ from ASV community structures

A total of 409 million metatranscriptomic 150 bp-long, quality-controlled paired-end reads were obtained from 25 gut samples (*n* = 5 per site; Table S1), which sufficiently covered approximate 97.6 ± 0.4% of the diversity in the samples (Fig. S2). Direct taxonomic annotation of these merged paired-end reads classified 2.35 ± 0.02% microbial reads (Table S1). This level of annotation is comparable with other studies in host-associated systems and most probably due to incomplete taxonomic coverage in reference databases and high levels of host nucleic acids [24, 33, 53]. Gene classification using marker ribosomal genes was not possible due to the rRNA depletion performed during library prep and subsequent biased removal of these common taxonomy markers [54]. Other commonly used methods based on house-keeping genes are not useful with environmental samples as the ones in this study, most probably due to the incomplete levels of annotation of these marker genes in these sample types [55]. Sixty-eight bacterial Orders across 29 Phyla were detected in the taxonomically annotated reads, of which 36 (53%) were also detected in the gut 16S amplicon data. The most active annotated phyla in the gut samples (all oysters) were *Proteobacteria* (46 ± 5%) and *Firmicutes* (16 ± 8%) (Fig. 4b). The most active taxa (*Bacillales*, *Pseudomonadales*, and *Enterobacterales*; as detected in the metatranscriptomes) were not the most abundant taxa (*Cyanobaceria, Mycoplasmatales*, and *Unknown Proteobacteria*; as detected by 16S rRNA gene amplicon analysis) (Fig. 4b). There were 103 (out of 296, 35%) Orders detected in the oyster gut 16S amplicons that were not detected in the metranscriptomes, most likely due to methodological biases (Figs. S2 and Fig. 4a).

While microbiome structures of oyster gut samples (detected by 16S rRNA gene amplicon analysis) showed differences between sites (Fig. 3b), microbiome structures of the active taxa (determined by taxonomic annotation of metatranscriptomic reads) in the gut samples were not different between sites (Fig. S5, species level). This may be due to the higher number of species detected using the metatranscriptomic approach as compared to the more conserved 16S rRNA gene amplicon sequencing, as seen by comparing the “core microbiome” detected with each method (taxa occurring in > 80% of samples, Fig. S6 and Table S3). Metatranscriptomic analysis detected a higher number of conserved species, as compared to the 16S rRNA gene amplicon analysis (72 conserved taxa in metatranscriptomes compared to 15 16S rRNA gene ASVs). Of those, only 2 taxa were identified by both methods, including a *Propionibacteriaceae* and a *Synechococcus* sp. The largest portion of the conserved sequences in both datasets corresponded to the Gammaproteobacteria, with 21 and 3 annotated taxa in the metatranscriptomic and 16S rRNA gene amplicon datasets respectively, followed by Actinobacteria (7 and 2 respectively). Gammaproteobacters conserved in oysters from Rhode Island waters included several Aeromonads, Enterobacters, Pseudomonads, and Vibrionales as identified by the metatranscriptomic dataset. Other conserved taxa uniquely detected by the metatranscriptomic dataset included annotations to Acidobacteria (1), Bacteroidetes (2), Chlamydia (1), Firmicutes (12 bacilllales and 5 clostridiales), Fusobacteria (1), Spirochaetaceae (1), and Verrumicrobiales (1). On the other hand, the 16S rRNA gene analysis detected one conserved ASV in the mollicutes (Fig. S6 and Table S3).

### Transcriptional responses in the oyster gut microbial community reflect the estuarine gradient in Narragansett Bay

**Fig. 5:**
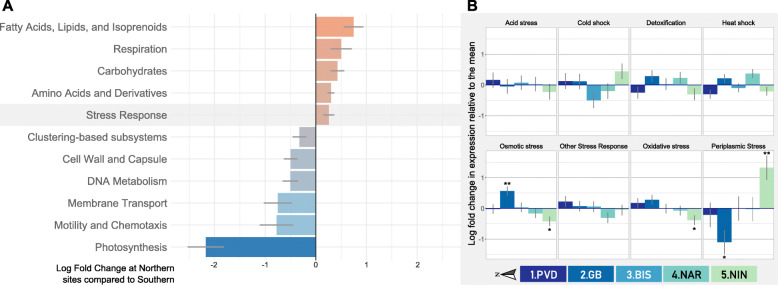
Pathways showing significant differential gene expression patterns between sites. **a** Differential expression of all significant (Benjamini-Hochberg *p*adj < 0.05) Level 1 pathways at the Northern sites (1–3, *n* = 15), compared to Southern sites (4–5, *n* = 10). A red bar (fold change> 0) indicates upregulation in the North and a blue bar (fold change< 0) indicates downregulation in the North. **b** Differential expression of Level 2 Stress Response pathways at each site, relative to the mean of all other sites (*n* = 5, Benjamini-Hochberg *padj < 0.05, **padj < 0.01)

Although no significant differences were detected between sites on the taxonomy of the transcriptionally active microbial taxa in the gut tissue (Fig. S5), their transcriptional responses varied based on the environmental conditions at each site (Fig. 5). In order to increase statistical power in the characterization of environment (i.e. eutrophication) on gene expression, the microbial transcriptional response at the more eutrophic/urbanized northern sites (1–3) was compared to that of the southern sites (4–5; considered as the less urbanized “control group”). This resulted in 11 SEED Level-1 pathways that showed significantly differential gene expression between northern and southern sites. A significant upregulation of bacterial stress responses and general metabolic activities (carbohydrates, respiration, amino acids, fatty acids, lipids etc.) was seen at northern sites, as well as a downregulation of photosynthesis, metabolic transport, and motility and chemotaxis (Benjamini-Hochberg adjusted *p* < 0.05; Fig. 5a). Select pathways were then further analyzed by comparing gene expression at each site to the mean of all sites.

#### Stress response

**Fig. 6:**
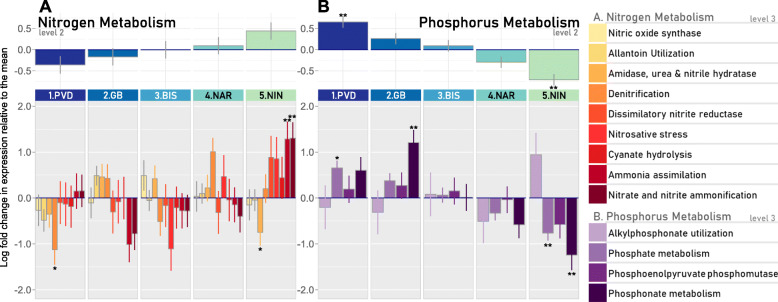
Differential gene expression in nitrogen and phosphorus metabolism at each site. **a** Differential expression of Nitrogen pathways at all sites, relative to the mean of the others (*n* = 5, Significance: Benjamini-Hochberg *padj < 0.05, **padj < 0.01). (top) Total differential expression of overall nitrogen metabolism, indicated with the blue-green colors. (bottom) Relative log fold change in nitrogen metabolism pathways, indicated with yellow-red colors. **b** Differential expression of Phosphorus pathways at all sites, relative to the mean. (top) Total differential expression of overall phosphorus metabolism, indicated with the blue-green colors. (bottom) Relative log fold change in phosphorus metabolism pathways, indicated with purple colors

In order to further examine the effects of anthropogenic factors (e.g. eutrophication, urbanization) on oyster microbial community and function, a more in-depth analysis of differences in the expression of genes involved in stress responses and nutrient cycling was performed (SEED level 2 annotation). Differential expression of genes in stress response and nutrient pathways at each of the sites was compared to the mean level of expression at all other sites (Figs. 5 and 6b). A significant upregulation in the expression of microbial genes involved in dealing with osmotic stress was detected in samples from 2.GB (as compared to the mean of all sites), as well as a significant downregulation in genes involved in periplasmic stress (*p <* 0.05). Conversely, a significant upregulation in genes involved in periplasmic stress (e.g. *rseA, degS, deQ*) and downregulation in genes involved in osmotic stress (e.g. genes coding for betaine aldehyde dehydrogenase and choline dehydrogenase) and oxidative stress (e.g. genes coding for NAD G3P dehydrogenase) was detected in oyster gut samples from 5.NIN *(p* < 0.05, Fig. 5b, S7, and S8). Expression of microbial genes involved in other acute stress responses, including acid stress, cold shock, and heat shock, were not significantly different between sites.

#### Nitrogen metabolism

Nutrient cycling is central to ecosystem services provided by oysters. Nitrogen and phosphorus are especially important, since they are the major components of eutrophication and often limiting factors to primary production [11, 56]. No significant changes in expression of genes involved in overall nitrogen metabolism (SEED level 2 annotation) was observed in the gut oyster microbiome from the different sites (Fig. 6a top), despite the significant differences in levels of nitrate, nitrite, and ammonium levels detected between sites (Table 1). High levels of variability in the expression of genes involved in the different pathways involved in nitrogen metabolism were observed between oysters within sites. Significant differences between sites were observed, however, in the patterns of expression of genes from specific pathways involved in nitrogen metabolism (SEED level 1 annotation; Fig. 6a bottom), reflecting differences in the responses of oyster gut microbes to the environmental conditions at each site. At the northernmost site (1.PVD), there was a significant downregulation of denitrification and NO detoxification-related genes (e.g. *nosF* and cytochrome c-dependent nitric oxide reductase (*cNor*)) compared to the mean of all sites, while at the southernmost site (5.NIN), a significant upregulation of genes involved in ammonia pathways (e.g. genes coding for NR(I), GNLE, and nitrate reductase) and a downregulation of nitrilase genes was observed (*p* < 0.05, Fig. S9).

#### Phosphorus metabolism

Expression of genes in the oyster gut microbiome involved in phosphorus metabolism decreased down the Bay, with microbial communities in the guts of oysters from the most southern (5.NIN) and northern (1.PVD) sites respectively showing significantly lower and higher levels of expression of genes involved in phosphorus metabolism than the mean of the sites (*p* < 0.01*;* Fig. 6b top). An upregulation of genes involved in the phosphate pathway (e.g. alkaline phosphatase) was observed in the gut microbiome of oysters from the northernmost site (1.PVD), as well as an upregulation of genes in the phosphonate pathway in oysters from 2.GB (e.g. phosphonoacetaldehyde hydrolase) (Fig. S6b bottom and S10). These two sites also showed the highest ambient phosphate concentrations (Table 1). Conversely, there was a significant downregulation of phosphate and phosphonate pathways at the southernmost site compared to the mean of all sites (5.NIN, *p* < 0.01, Fig. S6b bottom).

## Discussion

A better understanding of the effect of environmental conditions on both the structure and function of oyster-associated microbes is important for the management of oyster populations and optimization of the ecosystem services they provide. This study characterized the composition and function of eastern oyster-associated microbiomes at sites within a temperate, urbanized estuary. Oyster gut microbiomes during the summer were diverse in composition and differed between sites. Differences between the structure of microbiomes between water and oyster gut were consistent with selection and amplification of taxa from the water environment by the oyster host, as described in previous work [50]. Significant differences in expression of several gene pathways (stress response, nutrient utilization) were observed between sites, reflecting the environment at each of the sites. In particular, the gut microbial community of oysters collected at the northern sites, experiencing higher levels of nutrients and anoxia, showed upregulation of genes associated with stress response and phosphorus metabolism, as compared to the less eutrophic southern sites. Microbes in the gut of oysters from the less eutrophic sites showed a relative upregulation of genes associated with nitrogen metabolism. These responses varied according to the eutrophication gradient, indicating that the responses of oyster gut-associated microbiomes reflect the local environment, despite the fact that they are located within the host (i.e. the oxygen and nutrient status of the water is pervasive in the oyster gut). This is the first study combining 16S rRNA gene amplicon and metatranscriptomic data to look at both the composition and function of microbes associated with bivalves in an estuarine eutrophication gradient, with findings that are relevant to the development of restoration projects geared to maximize ecosystem services provided by oysters.

Surprisingly, expression of certain pathways involved in nitrogen metabolism in oyster-associated microbiomes was significantly higher at sites with the lowest levels of nutrients (NO_2_^−^, NO_3_^−^, NH_4_^+^) in the water at the southern range of the estuarine gradient than at the more eutrophic northern sites. These results are consistent with previous findings showing that oxygen conditions control nitrogen and phosphorus cycling in the sediments by limiting nutrient availability [57], with high dissolved oxygen concentrations in water promoting nitrogen removal [58]. This interaction between oxygen concentration (or redox state) and nitrogen metabolism has been well-documented in marine sediments: higher DO and low NO_3_^−^ concentrations stimulate denitrification, while the opposite occurs with high NO_3_^−^ concentrations [59, 60]. Our findings indicate that the environment in the oyster gut, as it relates to N and P cycling, reflects the overall environmental conditions at the site, consistent with expectations from sediment and water column observations. In the more eutrophic, anoxic, and acidic waters of the Providence River, where there is no available ammonia, oyster-associated microbiomes would upregulate pathways using the phosphate available from the sediment [61, 62]. Oysters in these northern sites may have been enriched in microbes adapted to prefer phosphate over nitrogen, due to selection driven by prevailing environmental conditions. Alternatively, higher levels of expression of genes involved in ammonia and nitrate utilization at the southern sites, in which less eutrophic conditions were found for nitrite, could reflect a physiological compensation driven by a need to scavenge the lower amounts of nitrate at these sites [63]. This is, however, not observed for phosphate. The potential similarity in function of microbes involved in N and P cycling between oyster gut and seawater/sediment may be explained by unique aspects of the physiology and anatomy of bivalves like oysters. Bivalves are ectotherms with an open circulatory system, and perform high levels of filtration when actively feeding [64, 65]. Therefore, we hypothesize that the particular nutrients (N, P compounds) and conditions (DO, pH) driving N and P cycling, must be, overall, similar in the oyster gut to the ambient water environment.

Microbiomes in the gut of eastern oysters collected at each of the sites also reflected potential stressors at each of the sites. For example, the increase in microbial oxidative stress observed at 1.PVD and 2.GB has been widely observed in microbial communities in response to anoxia, pollution, and toxins [66, 67]. The upregulation of periplasmic stress response (due to stressors within the inner bacterial membrane) observed in samples collected at site 5.NIN is likely coupled with increased nitrogen metabolism and transport [68–70]. In general, as eutrophic conditions worsen, bacteria will expend more energy on stress response and metabolic activities, a trend that has also been shown in marine sediment microbiomes [71, 72]. Other stressors, including pathogens, toxins, or chemical pollutants may have contributed to the differential expression in stress response pathway between sites and require further study.

Comparison of the microbial composition between water and oyster samples suggest that oysters select and amplify certain bacterial species through feeding selection, niche colonization, and/or evasion of immunity, as shown in oysters and other invertebrate host species [73, 74]. The fact that bacterial composition in gut samples does not completely reflect the community in the water samples may indicate that oysters amplify rare members in the water community and/or retain bacteria previously acquired through time horizontally from the water or vertically from parents. Consistent with the hypothesis of amplification, certain bacterial taxa that are known intracellular anaerobes were relatively more abundant in gut than water samples. These include members of the *Mycoplasmatales*, *Mollicutes*, and *Clostridiales*. *Mycoplasmatales* have been identified as common invertebrate symbionts and are avid biofilm-formers, allowing them to survive and replicate in the host [75, 76]. Besides these intracellular bacteria, *Proteobacteria* formed the most abundant and active phylum in the overall community, consistent with published literature in oysters [34, 48, 77]. High variability in the relative abundances of certain taxa (i.e. *Mycoplasmatales* or *Caulobacterales*) among oysters within sites suggests that host acquisition of bacteria from surrounding waters is shaped not only through exposure during feeding, but also factors like host health, characteristics, origin, and/or genetics [42, 78]. Further examination of within-site variability and its relationship with other host parameters (e.g. health and physiological status, genetics) may reveal how certain taxa are promoted in each oyster [79]. Decreased microbial diversity has been associated with health-compromised hosts, which may limit their plasticity and ability to respond to environmental change [48, 80]. Conversely, microbiomes of unhealthy hosts may be associated with an increase in diversity in tissues other than the usually more microbially diverse gut, as opportunistic and pathogenic bacteria proliferate in these tissues when compromised by disease [81, 82]. The interplay between the environment, oyster-associated microbiomes, and host health will be the focus of further study.

The differences in microbial composition observed in oyster gut samples between the 16S rRNA gene and the metatranscriptomics data were not unexpected, considering that metatranscriptomes reflect actively transcribing taxa, while the 16S rRNA gene amplicon method detects both live and recently dead microbes [83]. Although a small percentage of the metatranscriptomic reads were annotated, this method was able to capture the annotated microbial diversity at the species level. There are other potential technical biases that could have affected differences in microbial composition as determined by these two different methods, including PCR bias introduced in the process of amplification of the 16S rRNA gene portion, RNA degradation during storage, and issues related to uneven annotation between taxa [84, 85]. Although we have tried to minimize the impacts of these biases through the inclusion of several quality controls commonly used in microbiome research (e.g. mock communities, the use of technical and biological replicates and complementary analyses methods) [86], these biases should also be considered in the interpretation of these results. Despite technical challenges, comparisons between the 16S rRNA gene amplicon data with metatranscriptomic analysis of the oyster-associated microbial community, however, may provide some initial insights into identification of which of the core microbes show a symbiotic relationship with the oyster host, versus those that are transient food in the gut (i.e. accumulate in the oyster gut through association with food selectively ingested by oysters through filter feeding) [50, 87]. In particular, of the selected taxa shown to be relatively more abundant in the gut samples as compared to the water, a subset was detected to be transcriptionally active, particularly *Bacillales,* and *Vibrionales,* suggesting that these bacteria are not immediately digested in the gut or eliminated by the immune system. These results are consistent with the fact that these taxa are commonly cultured from oyster samples [48, 50, 88, 89]. Conversely, despite the high relative abundance of *Synechococcales* and other *Cyanobacteria* detected both in oyster gut samples and water from some of the sites through 16S rRNA gene amplicon sequencing, the gut metatranscriptomes did not show a relative enrichment in levels of expression of genes involved in recent photosynthesis, suggesting this higher abundance was transient and a reflection of recent feeding activity.

## Conclusions

This study has implications for quantification of ecosystem services provided by eastern oyster restoration and aquaculture. In Narragansett Bay, oyster fisheries were a dominant industry in the late 1880s, but a combination of pollution, overfishing, and dredging lead to the collapse of oyster populations in the 1940s [90]. In recent years, numerous efforts have been made to renew oyster reefs and restore their ecosystem services in Narragansett Bay. A common goal of oyster restoration projects is improvement of water quality by stimulation of environmental denitrification [39, 40]. Our findings support that removal of bioavailable nitrogen by denitrification, an important ecosystem service provided by oysters, declines in low oxygen, nutrient rich environments [62, 91, 92]. Enhanced denitrification would occur at high dissolved oxygen and nutrient rich environments, such as the conditions observed at 4.NAR during the summer. This implies that if the environmental microbial community does not have the genes necessary for the nitrogen pathway and/or the environmental conditions do not favor the process, then the addition of oysters to the site will not promote the ecosystem service. The prevailing environmental conditions and function of the resident environmental microbial community should be considered when selecting sites for oyster farming and restoration. In this study, 4.NAR and 5.NIN would provide the greatest return on investment for a restoration project, if only the benefits of denitrification are considered.

In summary, the estuarine gradient affected eastern oyster-gut associated microbial communities through changes in community composition, microbial stress responses, and microbial nutrient utilization. Combined, these results have implications for environmentally-driven changes in oyster microbial acclimation and potential ecosystem services. As the effects of estuarine acidification are expected to increase due to the combined effects of eutrophication, coastal pollution, and climate change, it is important to determine relationships between host health, microbial community structure, and environmental conditions. The results presented here form a baseline for future studies exploring how human-driven estuarine acidification affect overall oyster health and their associated ecosysem services.

## Methods

### Sample collection

Five sites were selected along Narragansett Bay, Rhode Island, USA and wild eastern oysters, *C. virginica*, were collected from the northern 4 sites, and farmed oysters were collected from 5.NIN (no wild oysters were found). Environmental data for temperature, pH, DO, salinity, and chlorophyll-*a* were collected using a YSI 6 Series Multiparameter Water Quality Sonde (Model 6920VS) every 30 s for 15 min at each site during the morning and afternoon hours on 1 day of the week of sampling, and averaged to account for diel variation in these parameters [93]. Sample collections were completed at low tide at each site from August 17–25, 2017 and consisted of oyster and water samples at each of the 5 sites with scientific collector’s permit #212 granted by the RI Department of Environmental Management. A total of 150 oysters (30 per site) were randomly collected within a 10 m^2^ transect at each of the 5 sites. On the day of collection, whole oysters (with shell) were weighed, shell width and length was measured, and samples of gut tissues (around 300 mg) were dissected and immediately preserved in RNAlater (Invitrogen) for RNA/DNA extractions. Preserved tissue samples were stored at − 80 °C until nucleic acid extractions. Up to 1 L of seawater was collected from the location and depth at which the oysters were collected in each site. Duplicate seawater samples were filtered using a peristaltic pump onto a 0.22 μm Sterivex filter (Millipore Sigma), filled with 2 mL of RNAlater, and then stored at − 80 °C until DNA extraction. An additional sample of seawater (30 mL) was filtered through a 0.22 μm syringe-top Polyether-sulfone (PES) filter and frozen at − 80 °C for nutrient analyses. Nutrient concentrations (nitrite, nitrate, ammonium, and phosphate) were analyzed in triplicate using a Lachat QuickChem QC8500 automated ion analyzer operated by the University of Rhode Island Marine Sciences Research Facility.

### Gut DNA and RNA extraction

Total nucleic acids were extracted from 150 to 200 mg of gut tissue (*n* = 10 random oysters per site; 50 total) using the Qiagen Allprep PowerViral DNA/RNA extraction kit with modifications as follows. The gut tissue sample was added directly to a 0.1 mm glass bead tube (Qiagen), along with 600 μL of Solution PV1 and 6 μL of sterile β-mercaptoethanol to minimize RNA degradation. The samples were subjected to bead beating for 5 min, followed by proteinase K digestion at 55 °C for 1 h in a shaker at 300 rpm. The supernatant was transferred to a new microcentrifuge tube and the protocol continued according to the manufacturer’s recommendations. Following nucleic acid extraction, the concentration was quantified using a Nanodrop 2000 instrument (ThermoFisher).

RNA purification from a 5 μL total nucleic acid aliquot was performed using the DNase Max I kit (Qiagen) according to the manufacturer’s protocol in a 50 μL reaction volume for 5 oyster gut samples per site. DNA purification of a 30 μL total nucleic acid aliquot was performed using an adapted version of the DNeasy PowerLyzer PowerSoil Kit. In brief, the total nucleic acids were transferred to a new 2 mL microcentrifuge tube, 1200 μL of Solution C4 was added, then vortexed. Next, 4 μL of RNase A solution was added to the sample and incubated for 2 min at room temperature. The treated DNA was loaded to a spin column, washed with Solution C5, and eluted in 50 μL of Solution C6. DNA and RNA concentrations were quantified with both a Nanodrop 2000 instrument (ThermoFisher) and Qubit Fluorometer High-Sensitivity reagents (Invitrogen).

### Seawater DNA extraction

Total DNA from water samples was extracted from the duplicate Sterivex filters using the Qiagen Allprep PowerViral DNA/RNA and DNeasy PowerLyzer PowerSoil kits with modifications as follows. The RNAlater was flushed out of the filters using a sterile syringe, and filters were rinsed with 2 mL of 1X sterile nuclease-free Phosphate Buffer Saline (PBS, pH 7.4, Invitrogen). Solution PV1 (1800 μL) and β-mercaptoethanol (18 μL) were added to the filter cartridge and incubated at 37 °C for 30 min. Next, 20 μL of proteinase K was added to the filter and digested at 55 °C for 1 h. The supernatant was flushed from the filter, and the protocol continued according to the manufacturer’s recommendations. DNA was purified from the entire total nucleic acid product and quantified using the methods described above.

### Nucleic acid amplification and sequencing

In order to obtain a comprehensive representation of the gut microbial community and their activities, 2 types of sequencing were performed: 16S rRNA gene amplicon of the V6 region (DNA, a measure of overall composition) and whole shotgun metatranscriptomes (RNA, a snapshot of functional activity at the time of collection) [94]. Amplicons of the V6 region of the 16S rRNA gene in the 50 gut DNA samples (10 per site), 5 water samples, a sample of a mock community (Zymo Research DNA standard I), and blank PCR control were prepared using 967F/1064R primers. A two-step PCR reaction using 300 ng of gut DNA or 10 ng of water DNA was performed in triplicate 33 μL reactions as previously described [95, 96]. The PCR products were analyzed with 75 bp paired-end sequencing to obtain overlapping reads on an Illumina MiSeq at the Genomics and Sequencing Center at University of Rhode Island.

The metatranscriptomic libraries were prepared from 2 μg of gut RNA (*n* = 5 randomly selected per site), fragmented at 500 nt using Covaris ultrasonification, and treated with the Illumina Ribo-Zero Gold rRNA Removal Epidemiology Kit prior to library prep to remove both host and bacterial rRNA. Illumina TruSeq PCR-free library kits were used to prepare the libraries, and then verified using both KAPA library quantification kits and Agilent Bioanalyzer. The resulting metatranscriptomic libraries were sequenced on an Illumina NovaSeq S4 to obtain 2 × 150 bp paired-end reads at the Yale Center for Genome Analysis.

### Processing and analysis of sequencing data

16S rRNA gene amplicon sequences were demultiplexed and quality filtered using DADA2 (v1.6.0) implemented in QIIME2 (v2018.4.0) with additional parameters --p-trunc-len-r 65 --p-trunc-len-f 76 --p-trim-left-r 19 --p-trim-left-f 19 to determine analysis sequence variants (ASVs) [97, 98]. All ASVs were summarized with the QIIME2 pipeline (v2018.4.0) and classified directly using the SILVA database (99% similarity, release #132) [99, 100]. Processed ASV and associated taxonomy data was exported as a count matrix for analysis in R (v3.4.1). Significant differences in Orders between sample types or gut samples at each site were calculated using linear discriminant analysis effect site (LEfSe) implemented on the Galaxy server [101, 102]. Non-bacterial and chloroplast sequences were then removed, and the data was normalized by percentage to the total ASVs in each sample for further dissimilarity metric analysis.

All descriptive and statistical analyses were performed in the R statistical computing environment with the *vegan* v2.5.5 and *phyloseq* v1.28.0 packages [103, 104]. Rarefaction curves and sequencing coverage estimates were generated using the rarecurve() and rareslope() commands with sample = [number of reads in smallest sample] in *vegan* v2.5.5 [105]. Simpson’s diversity values were calculated for each sample at the ASV level using the vegan package and analyzed using the non-parametric Kruskal–Wallis rank sum test in R. Non-metric dimensional analysis (NMDS) was used to determine the influence of sample type or field site on the ASV-level composition, implemented using vegan. The Bray-Curtis dissimilarity metric was calculated with k = 2 for max 50 iterations and 95% confidence intervals (standard deviation) were plotted. Statistical testing of beta-diversity was done using the PERMANOVA *adonis2* test implemented in *vegan* (method = “bray”, k = 2) [106, 107]. Within-site variability was calculated using the command vegdist (method = “bray”, k = 2) and the matrix was simplified to include samples compared within each site. Additional visualizations were computed using the *ComplexHeatmap* v3.9 and *UpSetR* v1.4.0 packages [108, 109].

Raw reads from the microbial community metatranscriptomes were first quality controlled with Trimmomatic software v0.36 with parameters PE -phred33 SLIDINGWINDOW:4:15 MINLEN:70 [110]. Metatranscriptomic analysis was performed using scripts from the SAMSA2 pipeline [111], with the following modifications. The quality-controlled paired-end reads were combined using PEAR v0.9.10 and then rogue rRNA reads were removed from the merged reads using SortMeRNA v2.1 [112, 113]. Taxonomic and functional annotation of the data were performed against RefSeq and SEED Subsystem databases, respectively, using DIAMOND v0.9.23 [114]. Core microbiomes were calculated using a custom script in R to determine which taxa (16S rRNA amplicons: ASVs; metatranscriptomes: species) were present in > 80% of the samples in each dataset.

Custom scripts using DESeq2 v1.14.1 were used to calculate differential expression between sites using the command DESeqDataSetFromMatrix(), with values transformed to account for differences in read abundance between samples [115]. The resulting changes in expression at all pathway levels were exported to R for analysis and visualization using *ggplot2* v3.2.1 and *cowplot* v1.0.0 [116–118]. Significant differences in gene expression are reported using Benjamini-Hochberg adjusted *p*-values. NMDS analysis was used to describe differences in gene expression between sites, and was calculated at the species and order level as described above. All processed sequencing files, bash scripts, QIIME2 artifacts, and Rscripts to reproduce the figures in the manuscript are available on Zenodo [119].

### Environmental statistical analysis

All statistical analyses of environmental and sequencing data were performed in R (v3.4.1 R Development Core Team, 2011) as follows. The environmental principal component analysis (PCA) was calculated using the prcomp (scale = TRUE) command implemented in base *stats* v3.6.1, and then plotted using autoplot() enabled by *ggfortify* v0.4.7 [120]. Significant differences in environmental parameters between sites were determined using all raw data subset by site and parameter. The data per site was compared using the compare_means() command from the *ggpubr* v0.2.2 package [121]. The method for each comparison was defined as “anova” for initial testing, then “t.test” for pairwise comparisons. Adjusted *p*-values were calculated using the Benjamini-Hochberg method by adding “p.adjust.method = BH” to the command [115].

## Supplementary Information

**Additional file 1: Figure S1.** (A) Percent abundances of the 10 most abundant phyla for 16S rRNA gene amplicon sequencing data by sample type and site. All other taxa are grouped into “Others.” Mock community control samples are shown at the left. (B) Percent abundance of the top 10 most abundant ASVs in the mock community samples. All other taxa are grouped into “Others.”

**Additional file 2: Figure S2.** Sequencing coverage analysis for 16S rRNA gene amplicon (left) and Metatranscriptomic samples (right). (A) Rarefaction curves for each sample colored by site for gut samples or water samples. The minimum sample size (raremax) is shown and indicated with a dashed line on each plot. (B) The slope calculated at the raremax for each sample is shown. (C) The estimated coverage (100–100*slope) for each sample is shown. Mean coverage and standard deviation for each method is shown in the bottom right.

**Additional file 3: Figure S3.** Number of bacterial Orders shared between the oyster gut and seawater 16S rRNA gene amplicons at each site (vertical bars). The total number of Orders found in each group is shown in the horizontal bar graph on the right. Intersections in gray denote comparisons that include the water samples.

**Additional file 4: Figure S4.** (A) Linear discriminant analysis Effect Size (LEfSe) analysis of bacterial Orders in seawater (*n* = 10), compared to gut samples (*n* = 50) in the 16S rRNA gene amplicons (one-against-all). (B) LEfSe analysis of bacterial Orders in gut samples at each site (*n* = 10) in the 16S rRNA gene amplicons (all-against-all). Only significantly increased taxa are shown. Significance was determined by LDA score > 2.0, alpha value = 0.05 for factorial Kruskal-Wallis test, and alpha value = 0.05 for pairwise Wilxocon test.

**Additional file 5: Figure S5**. NMDS plot visualizations of Bray-Curtis beta-diversity (*k* = 2) at the (A) Species and (B) Order levels for gut metatranscriptomic samples by Site. The ellipse lines show the 95% confidence interval (standard deviation). *p*-values indicate significance of grouping with *adonis2* Permutational Multivariate Analysis of Variance Using Distance Matrices test.

**Additional file 6: Figure S6.** Heatmap of taxa identified as the core bacterial community in the (A) 16S rRNA gene amplicon data and the (B) metatranscriptomic data. Green boxes indicate the presence of the core taxa in each sample per site. Core taxa was defined as occurring in > 80% of the samples per sequencing type.

**Additional file 7: Figure S7.** Differential expression (log fold change) of SEED Level 4 gene annotation of Oxidative Stress response groups at each site, relative to the mean of the others. All significantly regulated genes are outlined in red and annotated with an asterisk (*n* = 5, Benjamini-Hochberg *padj < 0.05, **padj < 0.01).

**Additional file 8: Figure S8.** Differential expression (log fold change) of SEED Level 4 gene annotation of Osmotic and Periplasmic stress response groups at each site, relative to the mean of the others. All significantly regulated genes are outlined in red and annotated with an asterisk (*n* = 5, Benjamini-Hochberg *padj < 0.05, **padj < 0.01).

**Additional file 9: Figure S9.** Differential expression (log fold change) of SEED level 4 gene annotation of nitrogen metabolism pathways at each site, relative to the mean of the others. All significantly regulated genes are outlined in red and annotated with an asterisk (*n* = 5, Benjamini-Hochberg *padj < 0.05, **padj < 0.01).

**Additional file 10: Figure S10.** Differential expression (log fold change) of SEED level 4 gene annotation of phosphorus metabolism pathways at each site, relative to the mean of the others. All significantly regulated genes are outlined in red and annotated with an asterisk (*n* = 5, Benjamini-Hochberg *padj < 0.05, **padj < 0.01).

**Additional file 11: Table S1.** Sequencing summary statistics, including the number of reads that passed quality control (QC) in each 16S rRNA gene amplicon and metatranscriptomic sample. No metatranscriptomes were sequenced for water samples.

**Additional file 12: Table S2.** Bray-Curtis beta-diversity summary statistics calculated using adonis2 Permutational Multivariate Analysis of Variance Using Distance Matrices test.

**Additional file 13: Table S3.** Taxa present in > 80% of all samples in the 16S rRNA gene amplicon and metatranscriptomic datasets. This table corresponds to Fig. S6.

**Additional file 14: Table S4.** Raw sequencing data accession information. This spreadsheet includes BioSample information for each oyster with corresponding Accession numbers for 16S rRNA gene amplicon or metatranscriptome files.

## Data Availability

The raw sequences generated for this study can be found in the NCBI Sequence Read Archive under BioProject no. PRJNA598635. Table S4 lists the corresponding BioSample and accession numbers for each individual sample. All processed sequencing files, bash scripts, QIIME2 artifacts, and Rscripts to reproduce the figures in the manuscript are available in the Zenodo repository, 10.5281/zenodo.3825254 [119].

## Abbreviations

PVD: Providence River, Bold Point Park
GB: Greenwich Bay, Goddard Memorial State Park
BIS: Bissel cove, Rome Point
NAR: Narrow River
NIN: Ninigret Pond
ASV: Amplicon sequence variant
BH: Benjamini-Hochberg
DO: Dissolved oxygen
LDA: Linear discriminant analysis
LEfSe: Linear Discriminate Analysis Effect Size
PBS: Phosphate buffer saline
PCA: Principal component analysis
PCR: Polymerase chain reaction
QIIME2: Quantitative Insights Into Microbial Ecology
RefSeq: Reference Sequence Database
SCC: Spearman’s correlation coefficient
