# Supplementary figures and images for: Functional plasticity in oyster gut microbiomes along a eutrophication gradient in an urbanized estuary
Source: Anim Microbiome. 2021 Jan 6;3:5. doi: 10.1186/s42523-020-00066-0 (PMC7934548; doi:10.1186/s42523-020-00066-0)

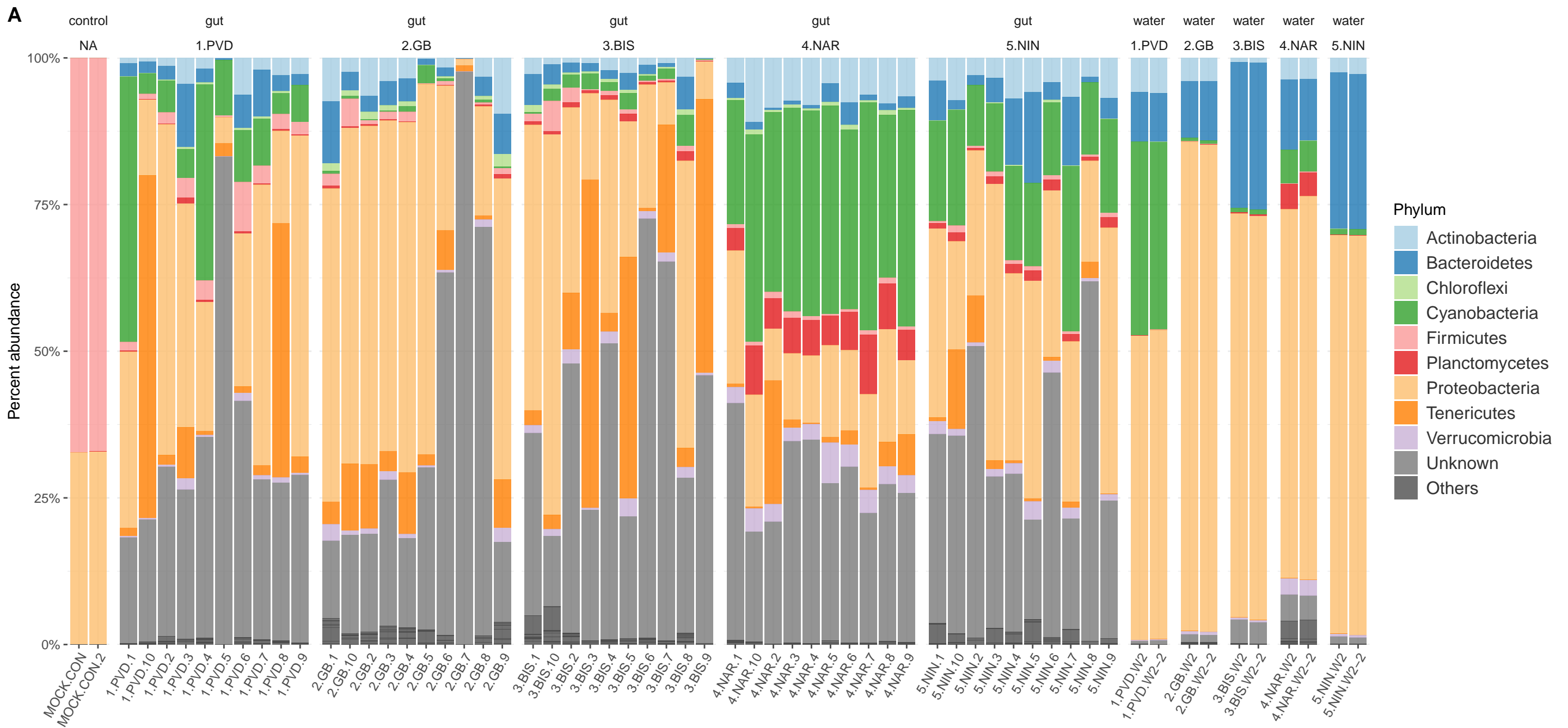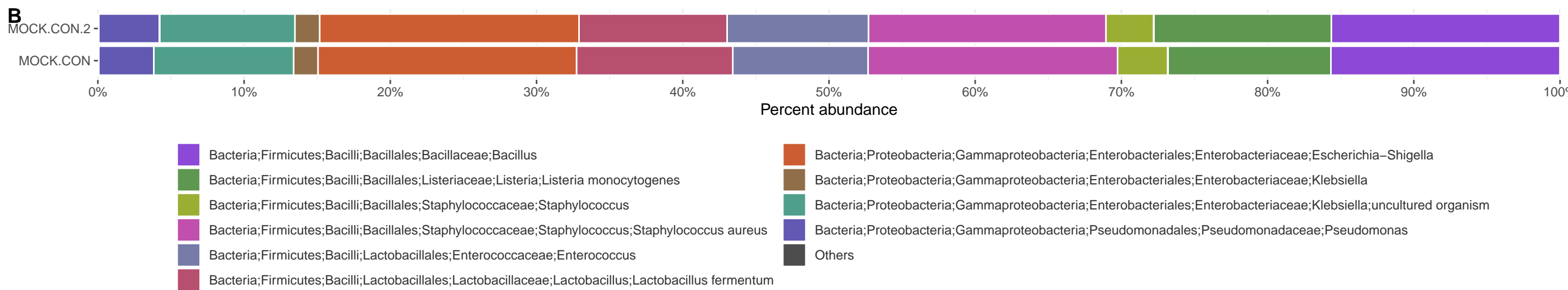

Supplement: Supplementary file 1 — Additional file 1: Figure S1. (A) Percent abundances of the 10 most abundant phyla for 16S rRNA gene amplicon sequencing data by sample type and site. All other taxa are grouped into “Others.” Mock community control samples are shown at the left. (B) Percent abundance of the top 10 most abundant ASVs in the mock community samples. All other taxa are grouped into “Others.” [file 42523_2020_66_MOESM1_ESM.pdf]

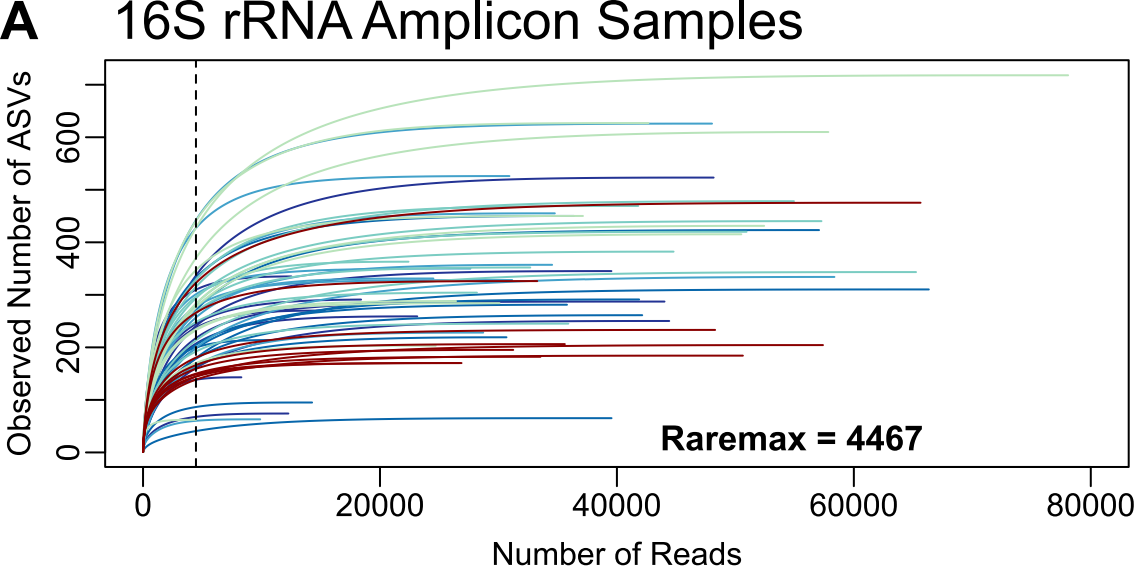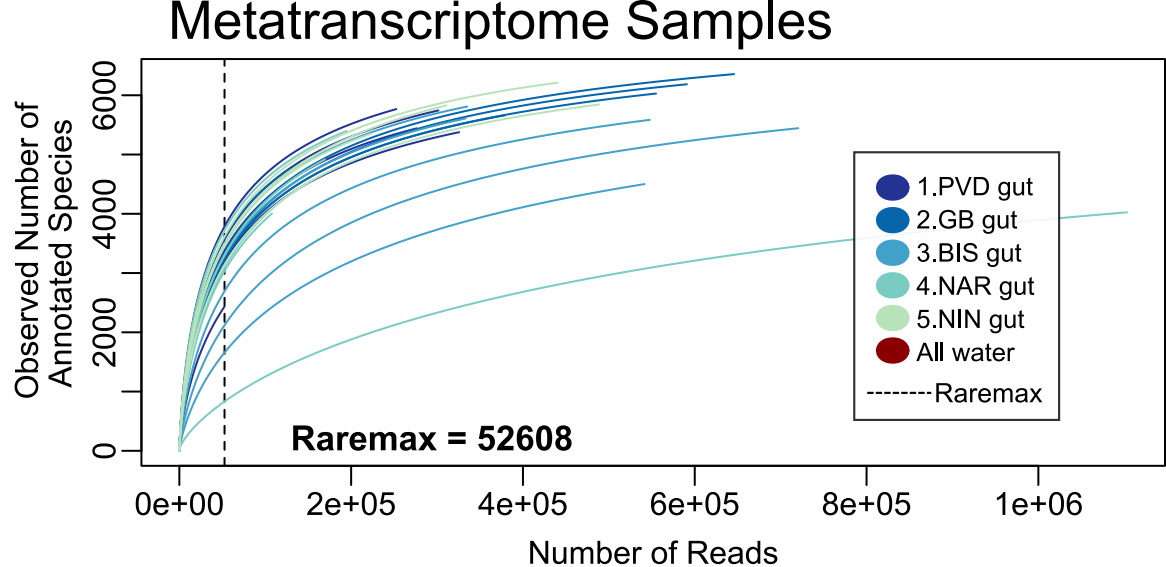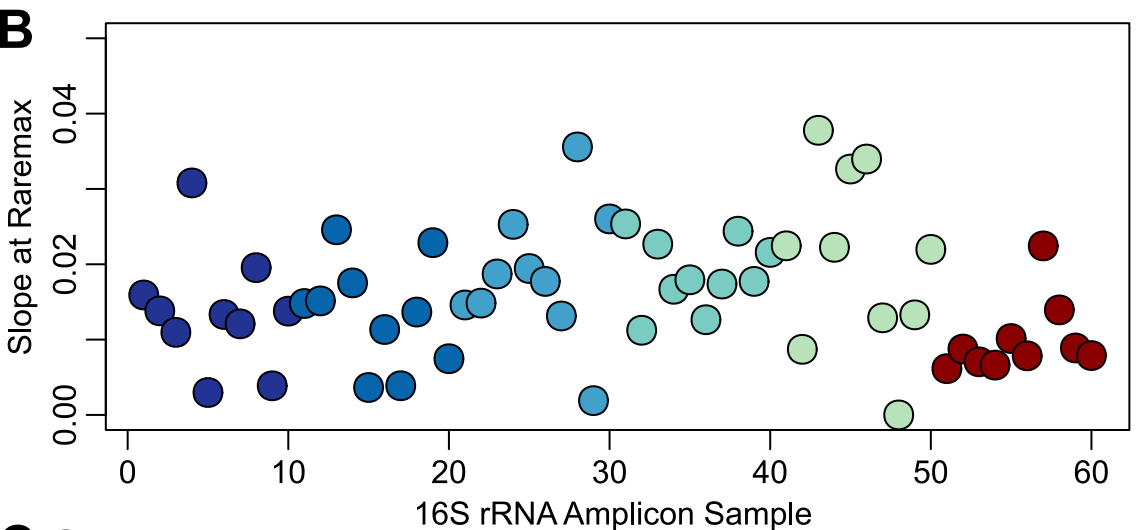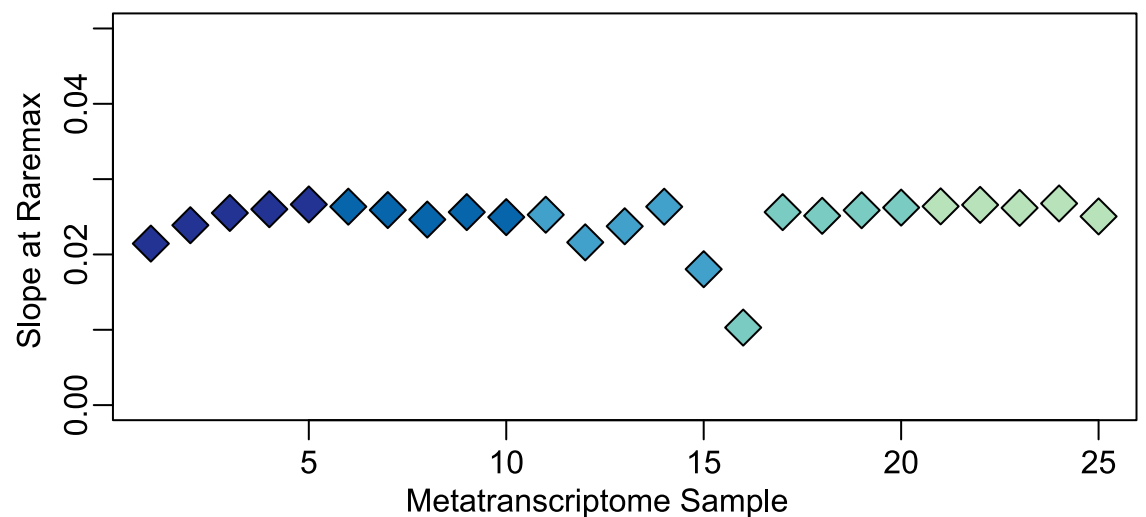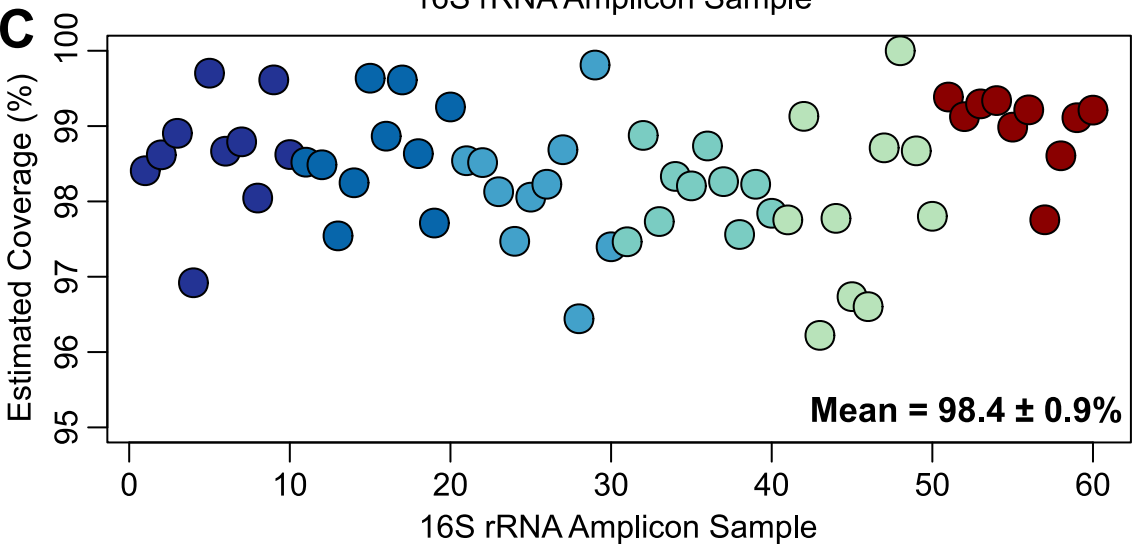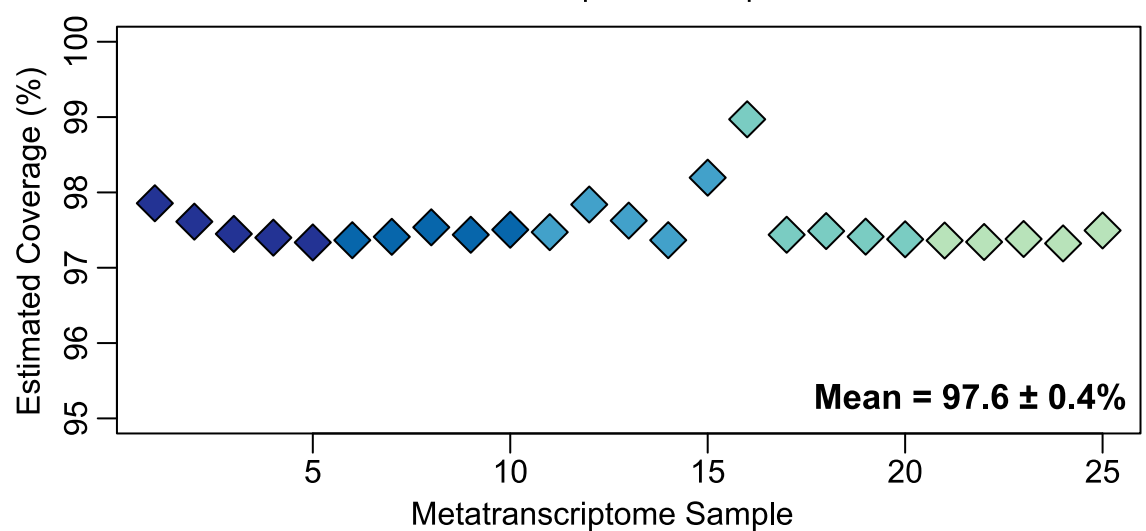

Supplement: Supplementary file 2 — Additional file 2: Figure S2. Sequencing coverage analysis for 16S rRNA gene amplicon (left) and Metatranscriptomic samples (right). (A) Rarefaction curves for each sample colored by site for gut samples or water samples. The minimum sample size (raremax) is shown and indicated with a dashed line on each plot. (B) The slope calculated at the raremax for each sample is shown. (C) The estimated coverage (100–100*slope) for each sample is shown. Mean coverage and standard deviation for each method is shown in the bottom right. [file 42523_2020_66_MOESM2_ESM.pdf]

Number of  
Orders  
in Common

80  
60  
40  
20  
0

Total Number of  
Orders per Group

0 50 100 150 200

All water 16S

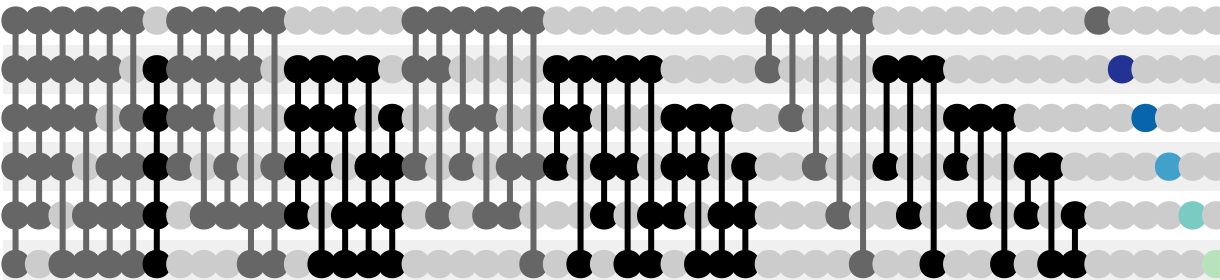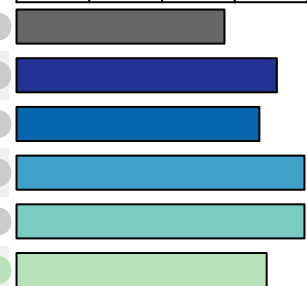

Supplement: Supplementary file 3 — Additional file 3: Figure S3. Number of bacterial Orders shared between the oyster gut and seawater 16S rRNA gene amplicons at each site (vertical bars). The total number of Orders found in each group is shown in the horizontal bar graph on the right. Intersections in gray denote comparisons that include the water samples. [file 42523_2020_66_MOESM3_ESM.pdf]

A

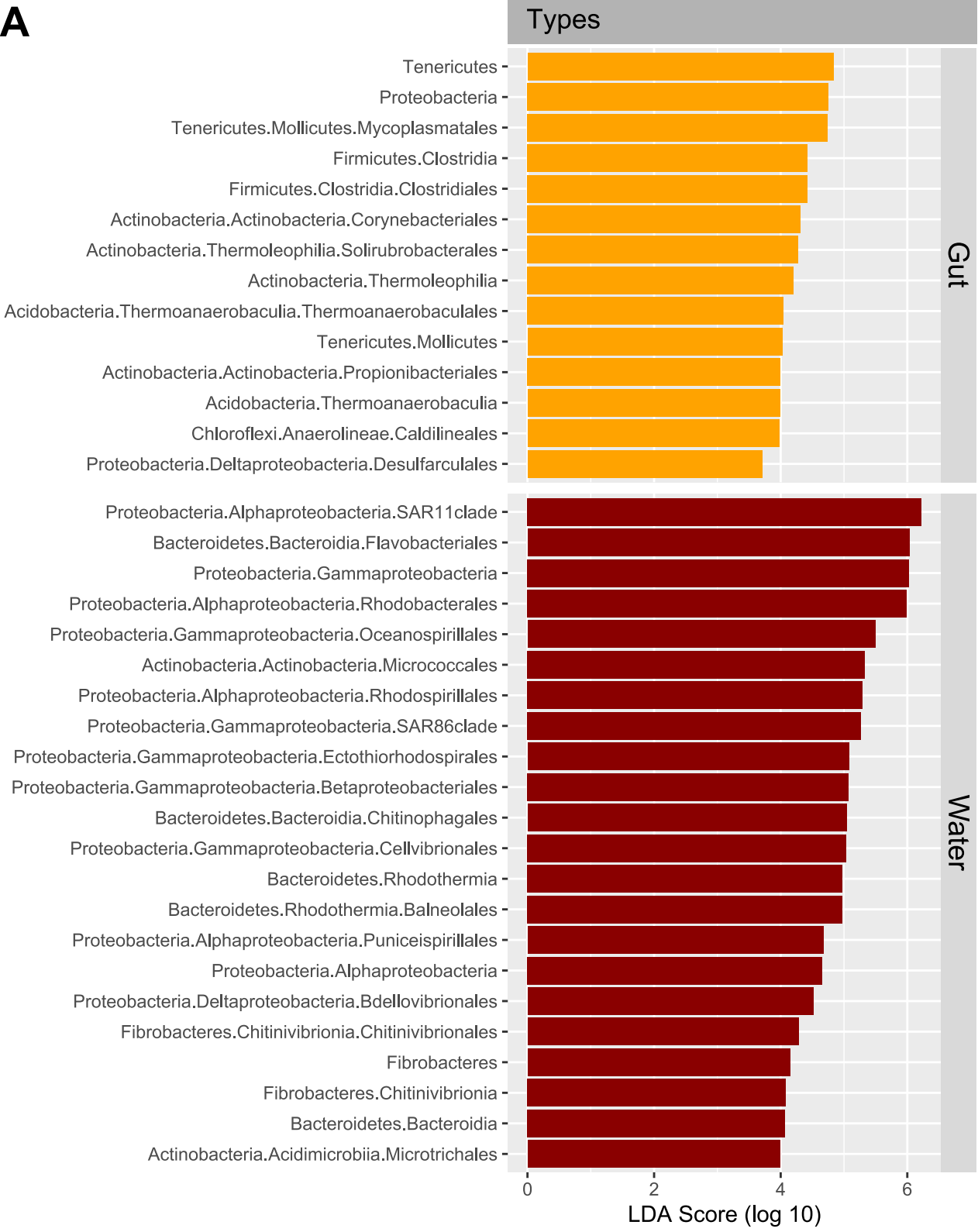

B

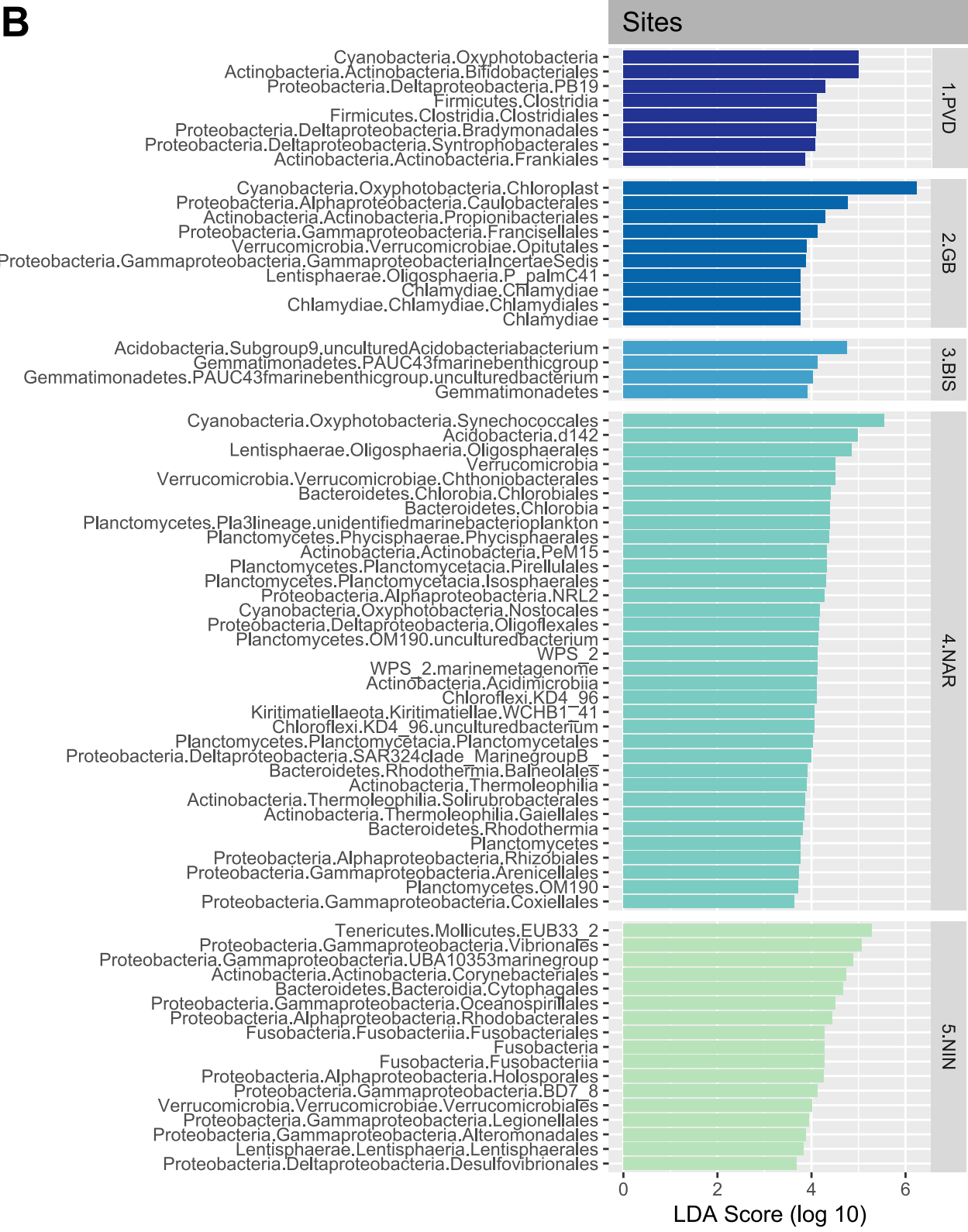

Supplement: Supplementary file 4 — Additional file 4: Figure S4. (A) Linear discriminant analysis Effect Size (LEfSe) analysis of bacterial Orders in seawater (n = 10), compared to gut samples (n = 50) in the 16S rRNA gene amplicons (one-against-all). (B) LEfSe analysis of bacterial Orders in gut samples at each site (n = 10) in the 16S rRNA gene amplicons (all-against-all). Only significantly increased taxa are shown. Significance was determined by LDA score > 2.0, alpha value = 0.05 for factorial Kruskal-Wallis test, and alpha value = 0.05 for pairwise Wilxocon test. [file 42523_2020_66_MOESM4_ESM.pdf]

## A Species level annotation

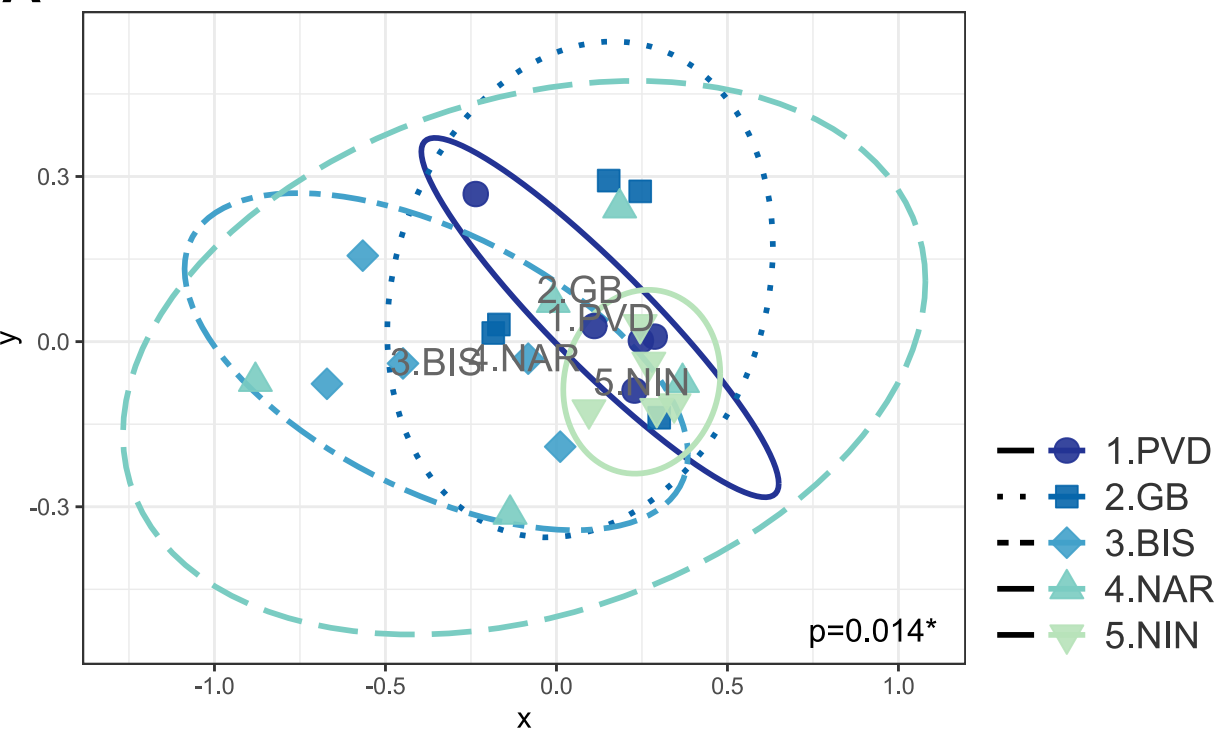

## B Order level annotation

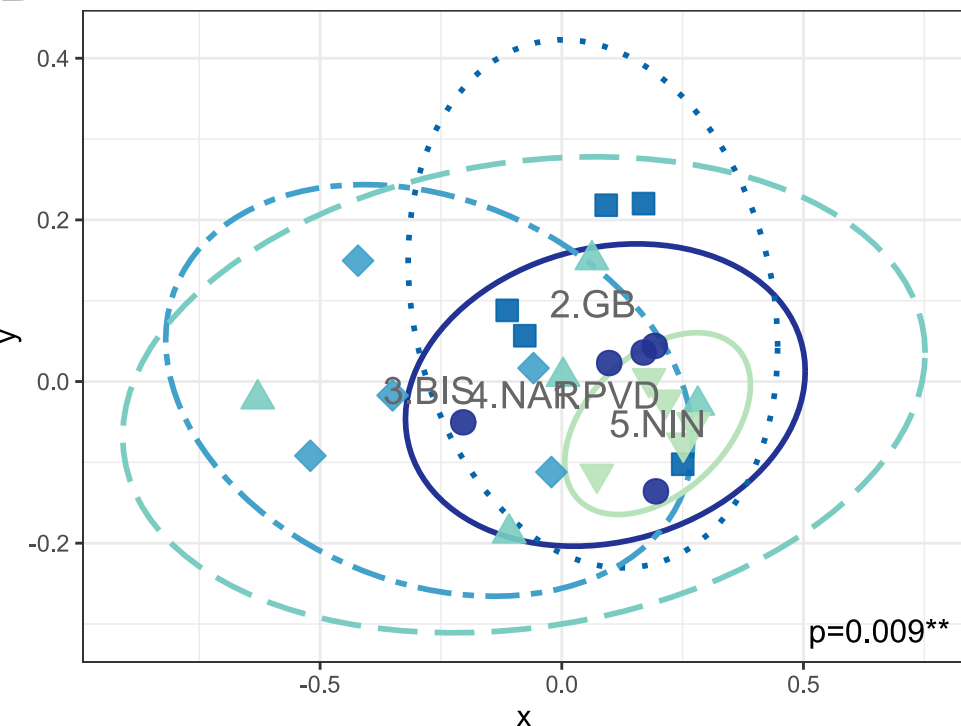

Supplement: Supplementary file 5 — Additional file 5: Figure S5. NMDS plot visualizations of Bray-Curtis beta-diversity (k = 2) at the (A) Species and (B) Order levels for gut metatranscriptomic samples by Site. The ellipse lines show the 95% confidence interval (standard deviation). p-values indicate significance of grouping with adonis2 Permutational Multivariate Analysis of Variance Using Distance Matrices test. [file 42523_2020_66_MOESM5_ESM.pdf]

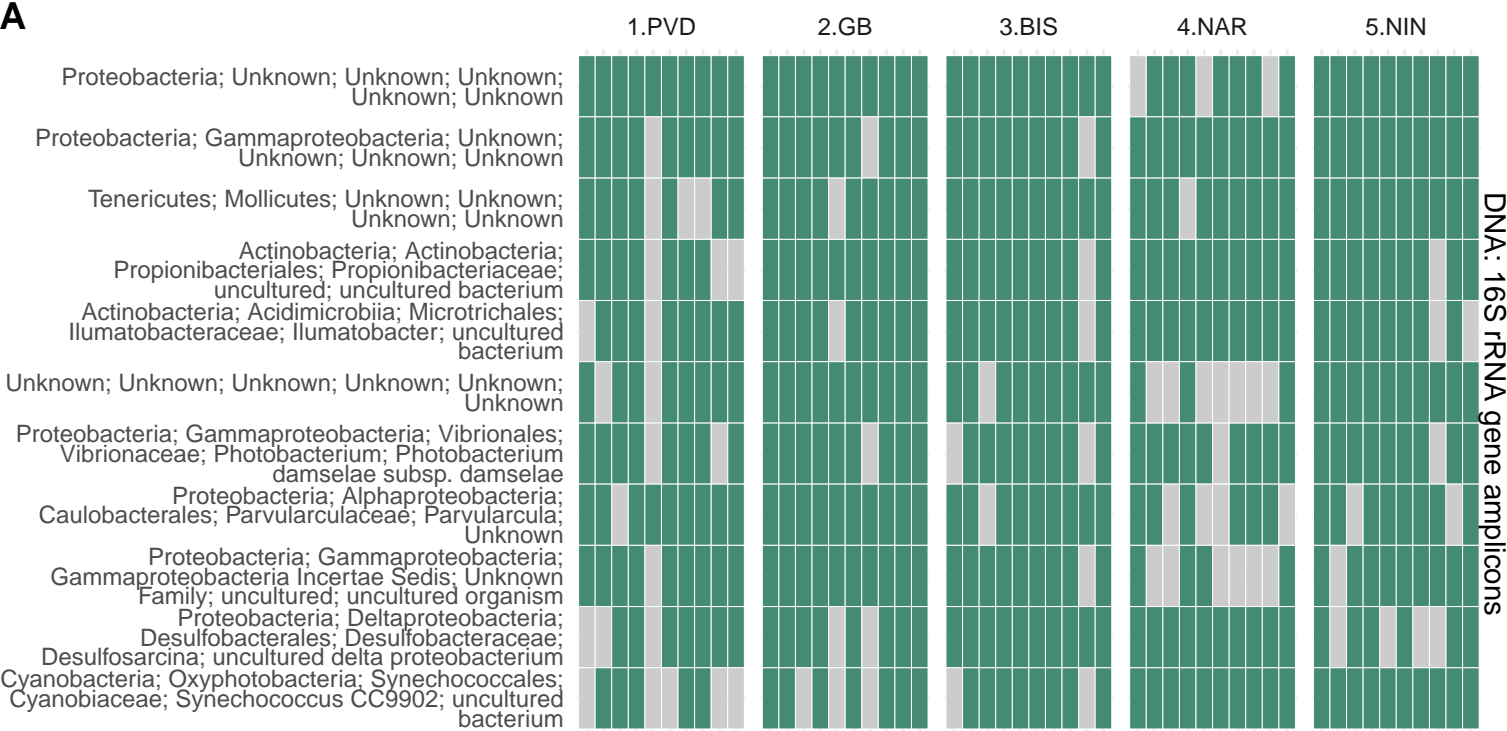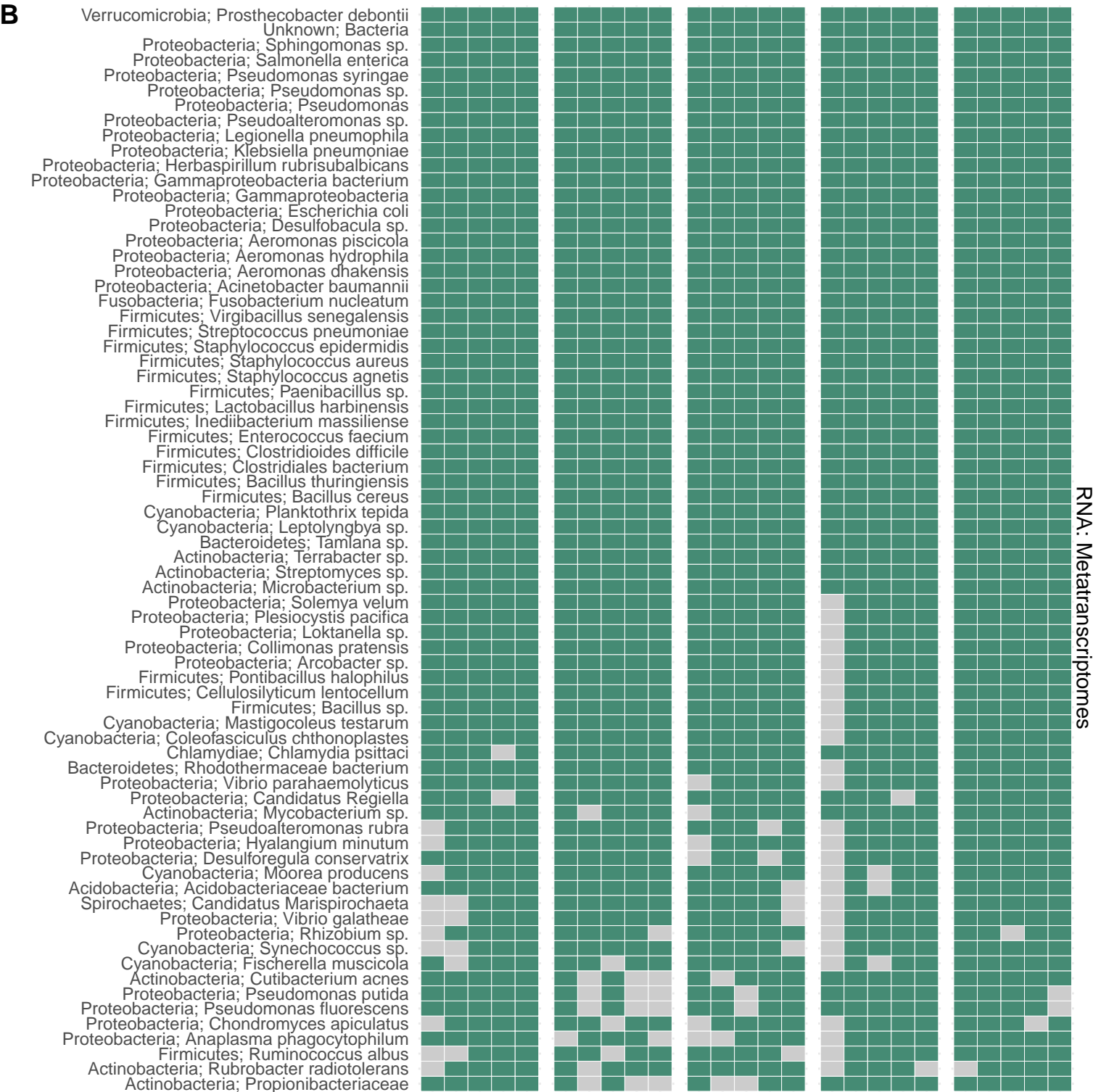

Supplement: Supplementary file 6 — Additional file 6: Figure S6. Heatmap of taxa identified as the core bacterial community in the (A) 16S rRNA gene amplicon data and the (B) metatranscriptomic data. Green boxes indicate the presence of the core taxa in each sample per site. Core taxa was defined as occurring in > 80% of the samples per sequencing type. [file 42523_2020_66_MOESM6_ESM.pdf]

## Osmotic Stress

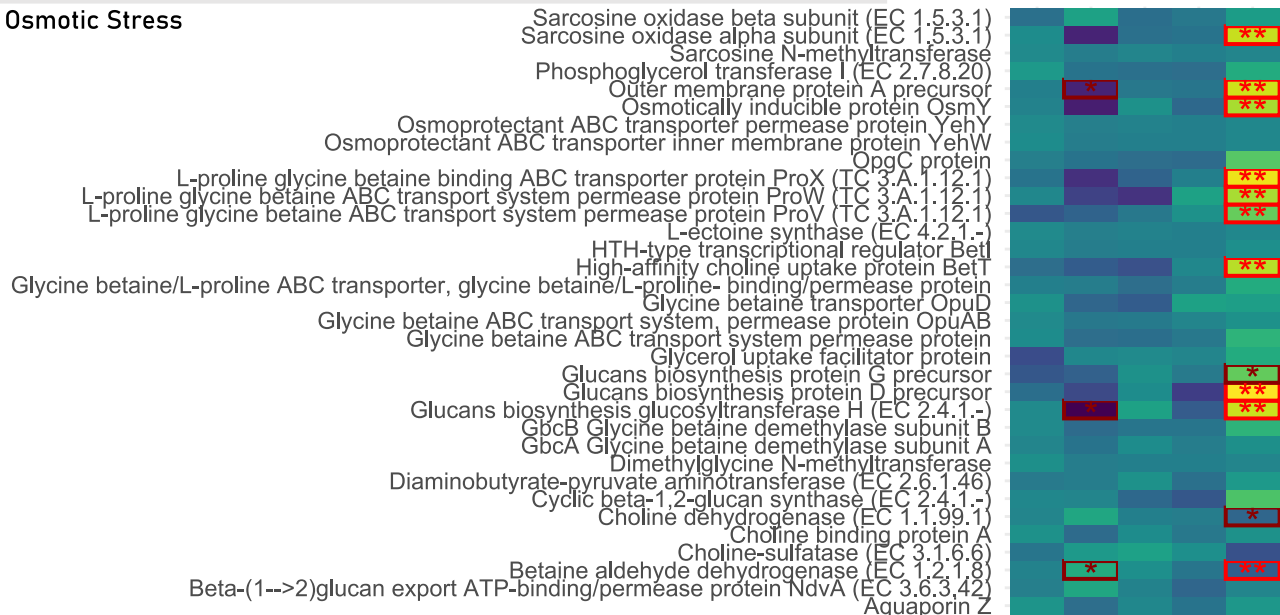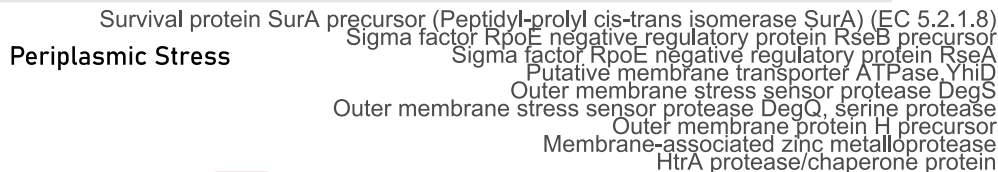

Padj  
value

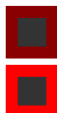

\* padj<0.05

\*\*padj<0.01

Log fold change in  
expression relative  
to the mean

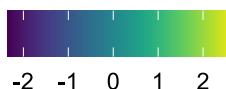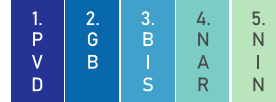

Supplement: Supplementary file 8 — Additional file 8: Figure S8. Differential expression (log fold change) of SEED Level 4 gene annotation of Osmotic and Periplasmic stress response groups at each site, relative to the mean of the others. All significantly regulated genes are outlined in red and annotated with an asterisk (n = 5, Benjamini-Hochberg *padj < 0.05, **padj < 0.01). [file 42523_2020_66_MOESM8_ESM.pdf]

# Nitrogen Metabolism

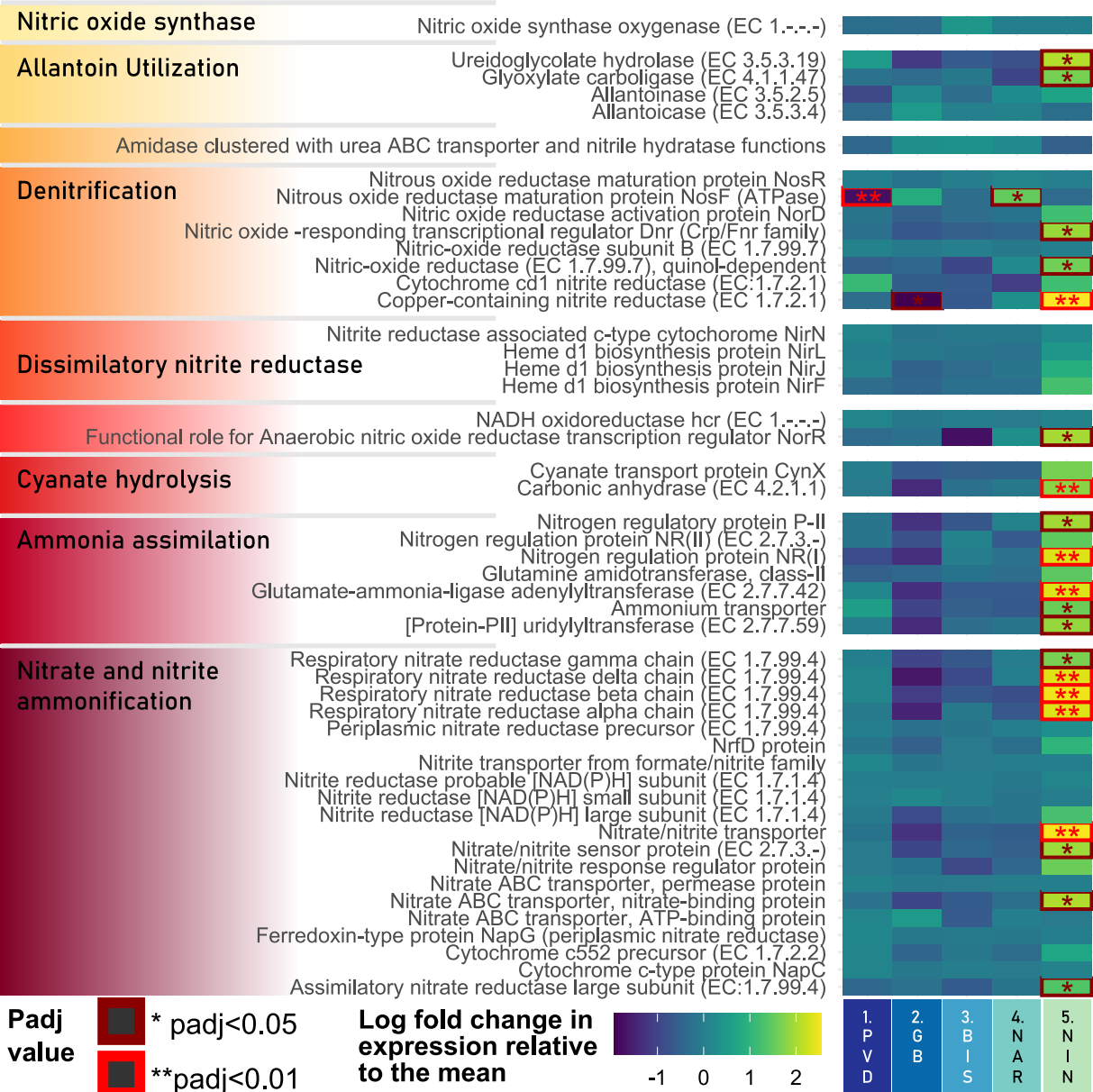

Supplement: Supplementary file 9 — Additional file 9: Figure S9. Differential expression (log fold change) of SEED level 4 gene annotation of nitrogen metabolism pathways at each site, relative to the mean of the others. All significantly regulated genes are outlined in red and annotated with an asterisk (n = 5, Benjamini-Hochberg *padj < 0.05, **padj < 0.01). [file 42523_2020_66_MOESM9_ESM.pdf]
