# Supplementary material for: Functional plasticity in oyster gut microbiomes along a eutrophication gradient in an urbanized estuary
Source: Anim Microbiome. 2021 Jan 6;3:5. doi: 10.1186/s42523-020-00066-0 (PMC7934548; doi:10.1186/s42523-020-00066-0)
Supplement: Supplementary file 7 — Additional file 7: Figure S7. Differential expression (log fold change) of SEED Level 4 gene annotation of Oxidative Stress response groups at each site, relative to the mean of the others. All significantly regulated genes are outlined in red and annotated with an asterisk (n = 5, Benjamini-Hochberg *padj < 0.05, **padj < 0.01). [file 42523_2020_66_MOESM7_ESM.pdf]

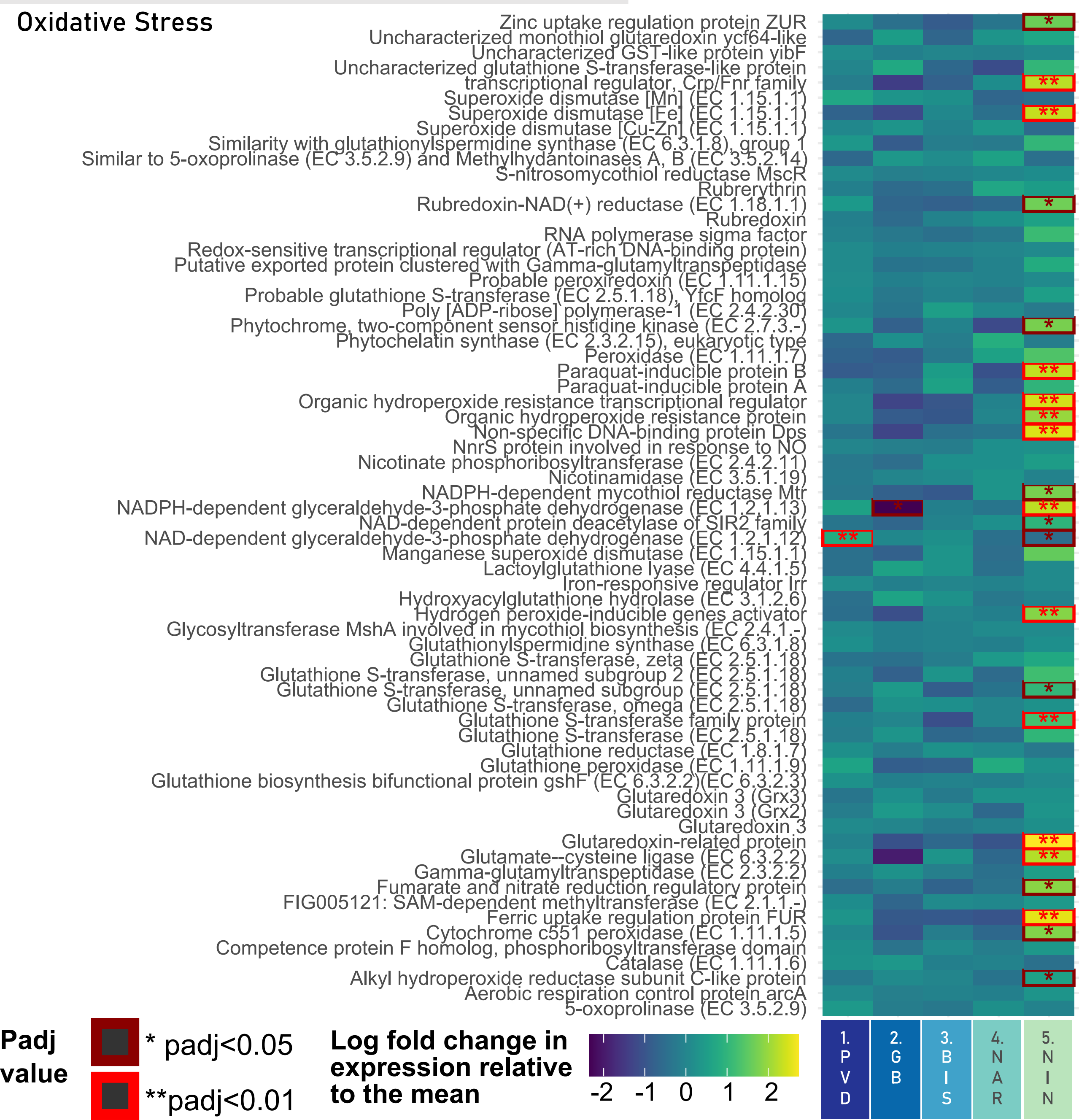

Oxidative Stress
Zinc uptake regulation protein ZUR
Uncharacterized monothiol glutaredoxin ycf64-like
Uncharacterized GST-like protein yibF
Uncharacterized glutathione S-transferase-like protein
transcriptional regulator, Crp/Fnr family
Superoxide dismutase [Mn] (EC 1.15.1.1)
Superoxide dismutase [Fe] (EC 1.15.1.1)
Superoxide dismutase [Cu-Zn] (EC 1.15.1.1)
Similarity with glutathionylspermidine synthase (EC 6.3.1.8), group 1
Similar to 5-oxoprolinase (EC 3.5.2.9) and Methylhydantoinases A, B (EC 3.5.2.14)
S-nitrosomycothiol reductase MscR
Rubrerythrin
Rubredoxin-NAD(+) reductase (EC 1.18.1.1)
Rubredoxin
RNA polymerase sigma factor
Redox-sensitive transcriptional regulator (AT-rich DNA-binding protein)
Putative exported protein clustered with Gamma-glutamyltranspeptidase
Probable peroxiredoxin (EC 1.11.1.15)
Probable glutathione S-transferase (EC 2.5.1.18), YfcF homolog
Poly [ADP-ribose] polymerase-1 (EC 2.4.2.30)
Phytochrome, two-component sensor histidine kinase (EC 2.7.3.-)
Phytochelatin synthase (EC 2.3.2.15), eukaryotic type
Peroxidase (EC 1.11.1.7)
Paraquat-inducible protein B
Paraquat-inducible protein A
Organic hydroperoxide resistance transcriptional regulator
Organic hydroperoxide resistance protein
Non-specific DNA-binding protein Dps
NnrS protein involved in response to NO
Nicotinate phosphoribosyltransferase (EC 2.4.2.11)
Nicotinamidase (EC 3.5.1.19)
NADPH-dependent mycothiol reductase Mtr
NADPH-dependent glyceraldehyde-3-phosphate dehydrogenase (EC 1.2.1.13)
NAD-dependent protein deacetylase of SIR2 family
NAD-dependent glyceraldehyde-3-phosphate dehydrogenase (EC 1.2.1.12)
Manganese superoxide dismutase (EC 1.15.1.1)
Lactoylglutathione lyase (EC 4.4.1.5)
Iron-responsive regulator Irr
Hydroxyacylglutathione hydrolase (EC 3.1.2.6)
Hydrogen peroxide-inducible genes activator
Glycosyltransferase MshA involved in mycothiol biosynthesis (EC 2.4.1.-)
Glutathionylspermidine synthase (EC 6.3.1.8)
Glutathione S-transferase, zeta (EC 2.5.1.18)
Glutathione S-transferase, unnamed subgroup 2 (EC 2.5.1.18)
Glutathione S-transferase, unnamed subgroup (EC 2.5.1.18)
Glutathione S-transferase, omega (EC 2.5.1.18)
Glutathione S-transferase family protein
Glutathione S-transferase (EC 2.5.1.18)
Glutathione reductase (EC 1.8.1.7)
Glutathione peroxidase (EC 1.11.1.9)
Glutathione biosynthesis bifunctional protein gshF (EC 6.3.2.2)(EC 6.3.2.3)
Glutaredoxin 3 (Grx3)
Glutaredoxin 3 (Grx2)
Glutaredoxin 3
Glutaredoxin-related protein
Glutamate--cysteine ligase (EC 6.3.2.2)
Gamma-glutamyltranspeptidase (EC 2.3.2.2)
Fumarate and nitrate reduction regulatory protein
FIG005121: SAM-dependent methyltransferase (EC 2.1.1.-)
Ferric uptake regulation protein FUR
Cytochrome c551 peroxidase (EC 1.11.1.5)
Competence protein F homolog, phosphoribosyltransferase domain
Catalase (EC 1.11.1.6)
Alkyl hydroperoxide reductase subunit C-like protein
Aerobic respiration control protein arcA
5-oxoprolinase (EC 3.5.2.9)
Padj value
* padj<0.05
**padj<0.01
Log fold change in expression relative to the mean
-2 -1 0 1 2
1. PVD
2. GB
3. BIS
4. NAR
5. NIN
