# Supplementary material for: Functional plasticity in oyster gut microbiomes along a eutrophication gradient in an urbanized estuary
Source: Anim Microbiome. 2021 Jan 6;3:5. doi: 10.1186/s42523-020-00066-0 (PMC7934548; doi:10.1186/s42523-020-00066-0)
Supplement: Supplementary file 10 — Additional file 10: Figure S10. Differential expression (log fold change) of SEED level 4 gene annotation of phosphorus metabolism pathways at each site, relative to the mean of the others. All significantly regulated genes are outlined in red and annotated with an asterisk (n = 5, Benjamini-Hochberg *padj < 0.05, **padj < 0.01). [file 42523_2020_66_MOESM10_ESM.pdf]

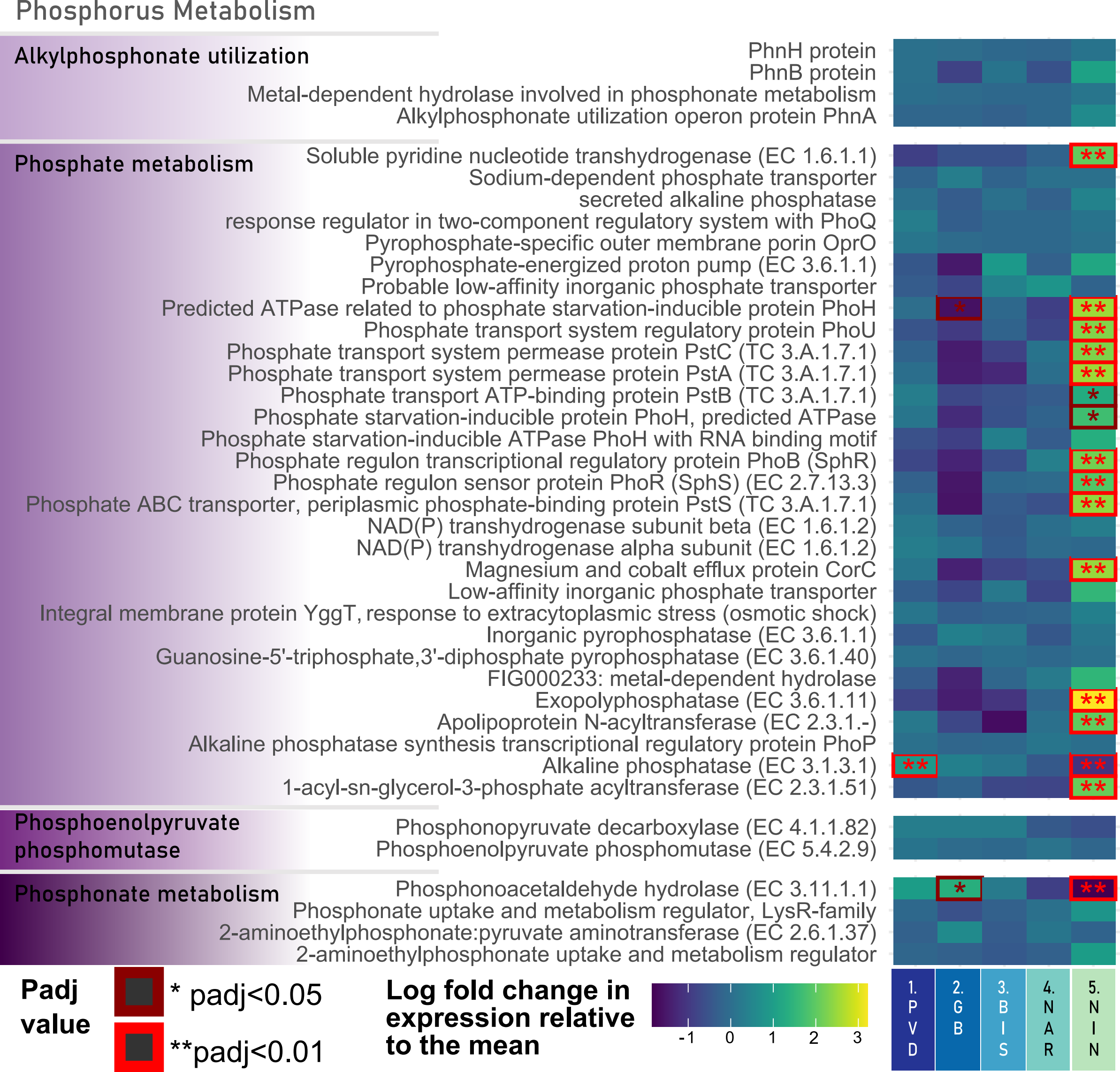
Phosphorus Metabolism
Alkylphosphonate utilization
PhnH protein
PhnB protein
Metal-dependent hydrolase involved in phosphonate metabolism
Alkylphosphonate utilization operon protein PhnA
Phosphate metabolism
Soluble pyridine nucleotide transhydrogenase (EC 1.6.1.1)
Sodium-dependent phosphate transporter
secreted alkaline phosphatase
response regulator in two-component regulatory system with PhoQ
Pyrophosphate-specific outer membrane porin OprO
Pyrophosphate-energized proton pump (EC 3.6.1.1)
Probable low-affinity inorganic phosphate transporter
Predicted ATPase related to phosphate starvation-inducible protein PhoH
Phosphate transport system regulatory protein PhoU
Phosphate transport system permease protein PstC (TC 3.A.1.7.1)
Phosphate transport system permease protein PstA (TC 3.A.1.7.1)
Phosphate transport ATP-binding protein PstB (TC 3.A.1.7.1)
Phosphate starvation-inducible protein PhoH, predicted ATPase
Phosphate starvation-inducible ATPase PhoH with RNA binding motif
Phosphate regulon transcriptional regulatory protein PhoB (SphR)
Phosphate regulon sensor protein PhoR (SphS) (EC 2.7.13.3)
Phosphate ABC transporter, periplasmic phosphate-binding protein PstS (TC 3.A.1.7.1)
NAD(P) transhydrogenase subunit beta (EC 1.6.1.2)
NAD(P) transhydrogenase alpha subunit (EC 1.6.1.2)
Magnesium and cobalt efflux protein CorC
Low-affinity inorganic phosphate transporter
Integral membrane protein YggT, response to extracytoplasmic stress (osmotic shock)
Inorganic pyrophosphatase (EC 3.6.1.1)
Guanosine-5'-triphosphate,3'-diphosphate pyrophosphatase (EC 3.6.1.40)
FIG000233: metal-dependent hydrolase
Exopolyphosphatase (EC 3.6.1.11)
Apolipoprotein N-acyltransferase (EC 2.3.1.-)
Alkaline phosphatase synthesis transcriptional regulatory protein PhoP
Alkaline phosphatase (EC 3.1.3.1)
1-acyl-sn-glycerol-3-phosphate acyltransferase (EC 2.3.1.51)
Phosphoenolpyruvate phosphomutase
Phosphonopyruvate decarboxylase (EC 4.1.1.82)
Phosphoenolpyruvate phosphomutase (EC 5.4.2.9)
Phosphonate metabolism
Phosphonoacetaldehyde hydrolase (EC 3.11.1.1)
Phosphonate uptake and metabolism regulator, LysR-family
2-aminoethylphosphonate:pyruvate aminotransferase (EC 2.6.1.37)
2-aminoethylphosphonate uptake and metabolism regulator
Padj value
* padj<0.05
**padj<0.01
Log fold change in expression relative to the mean
-1
0
1
2
3
1. PVD
2. GB
3. BIS
4. NAR
5. NIN
